# Chemical Recycling of Polycarbonate Acrylonitrile Butadiene Styrene Blends via Organocatalyzed Acetolysis

**DOI:** 10.1002/cssc.202502161

**Published:** 2026-01-05

**Authors:** Mary E. Pool, Edward Savage, Rachel Holland, Ciaran W. Lahive, Michael P. Shaver

**Affiliations:** ^1^ Department of Materials The University of Manchester Manchester UK; ^2^ Polestar Automotive UK Ltd Envisage, Unit F Coventry UK

**Keywords:** depolymerization, kinetics, multimaterials, polycarbonates, recycling

## Abstract

Polycarbonate acrylonitrile butadiene styrene (PC/ABS) is one of the most widely used plastic blends, with growing importance in both automotive and electronics applications. However, its heterogeneous nature disables recycling, leading to its disposal via landfilling or incineration. This work proposes a way to recycle this material via selective chemical recycling whereby the PC is depolymerized by acetolysis, heating the blend with acetic acid and a basic organocatalyst, leaving the ABS untouched. Catalytic optimization on PC feedstocks revealed that successful organocatalysts required not only sufficient basicity but also a basic nitrogen incorporated within an aromatic ring. A kinetic study revealed the depolymerization was pseudo first‐order with an activation energy of 96.7 kJ mol^−1^. Selective acetolysis was developed for both PC/ABS pellets and a PC/ABS automotive part. Separation of the PC monomers from the ABS was achieved with dialysis, with isolated ABS having similar properties to virgin grades. This approach offers a promising route toward recovering value from recalcitrant PC/ABS blends by enabling selective deconstruction of PC and recovery of ABS, thereby minimizing dependence on virgin plastic production.

## Graphical abstract

Polycarbonate/acrylonitrile butadiene styrene (PC/ABS) has been chemically recycled by depolymerizing the PC via base‐catalyzed acetolysis to yield acetylated bisphenol A. Unlike alternative methods, the presence of ABS did not hinder acetolysis, allowing recovery of ABS from these multimaterials. A new depolymerization catalyst, 4‐pyrrolidinylpyridine, was used, giving pseudo first‐order reaction kinetics with an activation energy of 97 kJ mol^−1^.

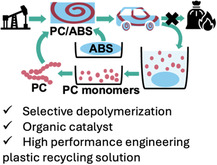

## 1 Introduction

The increasing use of plastics in vehicles has led the automotive industry to become a significant contributor to plastic waste [1, 2, 3]. This use continues to grow thanks to a typically high durability, low cost, and high strength‐to‐weight ratio [4]. The high specification and commingling of plastics in the transport sector, while necessary to meet stringent safety and structural requirements, presents a challenge at end‐of‐life (EoL) as they often exist as composites, laminates, or blends [4]. Mechanical recycling struggles with blended or mixed feedstocks, contributing to low EoL automotive plastics recycling rates (EU, 19%, 2023) [1], despite legislative efforts to promote it [5].

**FIGURE 1:**
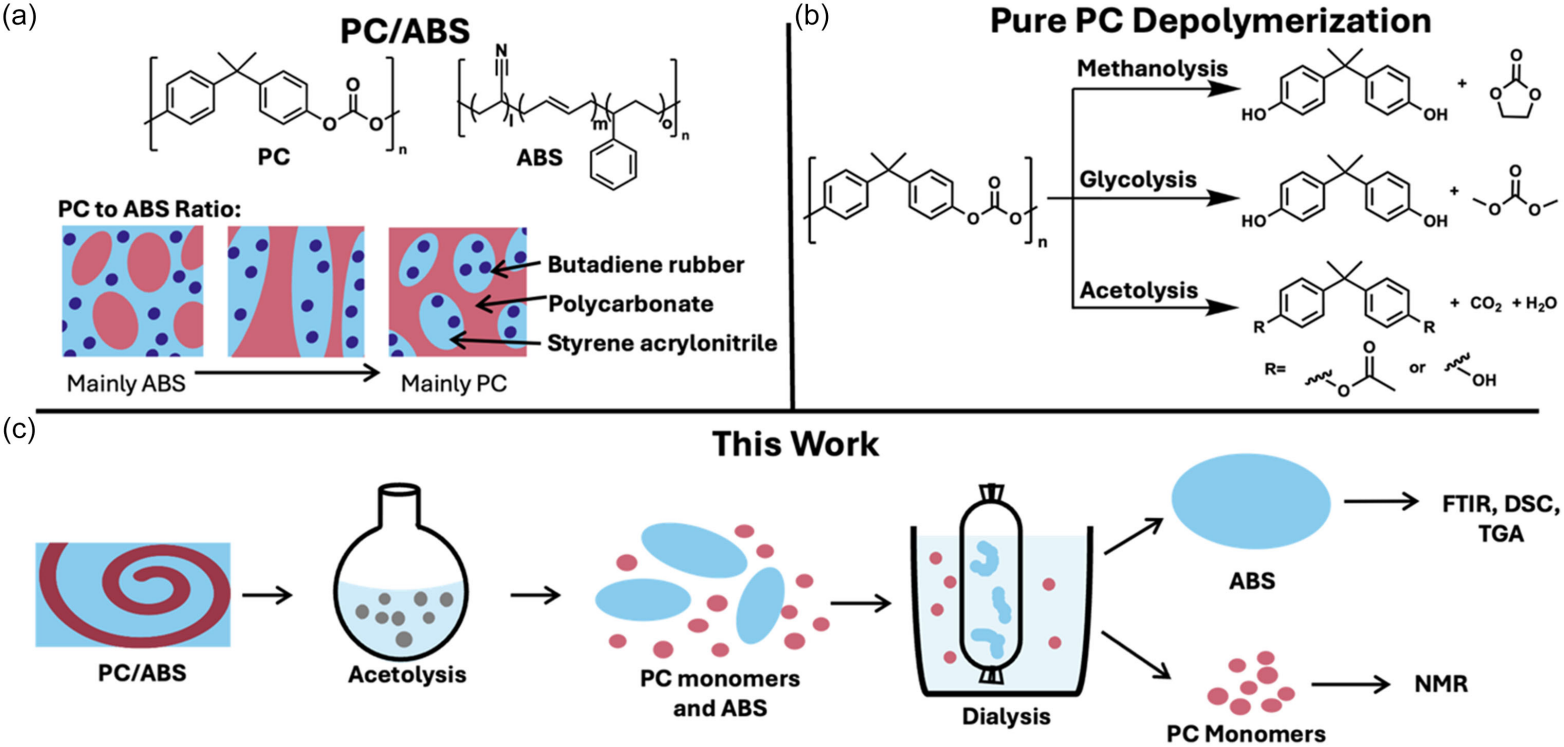
(a) The chemical structure of PC/ABS and a schematic illustrating the material's multiphase character, (b) PC depolymerization methods, and (c) a schematic of this work.

Polycarbonate/acrylonitrile butadiene styrene (PC/ABS), an engineering polymer blend widely used in the automotive industry (Figure 1a), has a particularly high environmental impact, largely due to the significant emissions involved in the constituent monomer synthesis of bisphenol A (BPA) [6]. Mechanically recycling PC/ABS is extremely challenging due to high feedstock heterogeneity, differing component ratios and additive formulations, ultimately leading to inconsistent and unpredictable recyclate properties [7]. Even closed‐loop, postindustrial reprocessing causes significant degradation unless high quantities of virgin material are added [8], while additionally posing a risk of forming leachable degradation products including the notably hazardous BPA [9].

Recovery of BPA from these systems is thus economically and environmentally important. Chemical recycling via depolymerization has been proposed as a method to bypass the material degradation caused during mechanical recycling and allow recycling of composite and blended materials [10, 11, 12, 13]. Depolymerization is particularly advantageous for carbonyl‐containing polymers, like polycarbonates, polyamides, and polyesters, where selective cleavage allows for the formation of high‐value products [10]. PC in isolation is most commonly depolymerized using techniques such as methanolysis, glycolysis, and hydrolysis which enable recovery of high‐value monomers such as BPA and carbonates (Figure 1b) [14].

There is limited literature surrounding the chemical recycling of PC/ABS and it primarily focusses on pyrolysis, a nonselective technique which produces a wide range of solids, liquids, and gases which require costly separation to enable further use [15, 16, 17]. The selective depolymerization of PC in PC/ABS blends is rare with just three studies published to date, exploring NaOH‐catalyzed methanolysis [18, 19] and Ni‐catalyzed reductive elimination [20]. Methanolysis of EoL PC/ABS required a reaction time 10 times greater than that for PC material, as the ABS hindered methanol ingress [18]. After 5 h at 175°C, the BPA yield was only 10% and BPA was found trapped in the residual ABS fraction [19]. An alternative method required separating the PC from the PC/ABS blend and removing any colorants or additives prior to PC depolymerization [18]. This procedure using activated charcoal in THF, centrifugation and reprecipitation produced a pure PC suitable for methanolysis [18] or reductive elimination [20]. Together, these examples highlight that there is scope for the development of new PC/ABS recycling processes that degrade PC into BPA without prior purification and with similar efficiency to pure PC. An additional aspect that has yet to be examined in the existing studies is the fate of the ABS and its potential to be recycled, representing a promising area for further investigation.

Acetolysis is an emerging depolymerization approach which has shown promise in selectively breaking down polymers within mixed materials such as epoxy‐amine carbon fiber reinforced polymers and poly(ethylene terephthalate) textiles [11, 21]. Previous acetolysis studies use catalyst‐free systems at high temperatures (280°C) with concomitant high operational pressures and energy usage [11, 21]. We hypothesized that organocatalysts would help alleviate these safety and sustainability challenges, while also facilitating the depolymerization of complex blends better than alcoholysis routes. Alberti et al. developed an acetic anhydride depolymerization of PC monomaterials [22]. The article also showed that catalytic acetic acid depolymerization was possible using microwave heating (180°C) to produce a mixture of BPA and mono‐acetylated (MA‐) and di‐acetylated (DA‐) BPA, CO_2_, and water (Figure 1b). The primary focus on acetic anhydride and monomaterials left much to explore. Herein, a method to depolymerize the PC within PC/ABS and recover both BPA derivatives and ABS was developed and exemplified on both blends and an automotive part (Figure 1c).

## 2 Experimental

### 2.1 Materials

All chemicals were purchased from Sigma‐Aldrich, Honeywell, TCI, VWR, or Fisher and used as received or as described below. The PC resin feedstock was TRIREX 3025N2, PC/ABS resin was uncolored Bayblend T65 XF, ABS resin was Terluran GP‐22, and the automotive part was a Polestar 3 air vent supplied by Polestar. Dialysis tubing was Snakeskin 68035 with a molecular weight cut off of 3500 Da. Kinesis 20 ml microwave vials, which were suitable to withstand the pressure build up during reactions, were used for depolymerization reactions.

### 2.2 Depolymerization Procedures

All plastic resins were dried in a vacuum oven at 60°C prior to being used. PC pellets (0.3 g) were added to a 20 ml crimp top vial containing a stirrer bar with the stated amount of acetic acid and catalyst. The vial was sealed then the headspace purged with nitrogen using a needle through the vial's septum for 2 min. The needle was removed, and the vial was placed in a preheated oil bath and stirred. After the required reaction time all vials were cooled to room temperature then depressurized by inserting a needle before removing the vial cap. For PC/ABS pellets, 0.5 g or 1.5 g of PC/ABS pellets were added, and the remainder of the procedure was unchanged. For the air vent, the part was cut into pieces of a similar size to pellets, and 0.5 g was added; the PC content was 72% of the mass of the air vent. The remainder of the procedure was unchanged. The high temperatures and CO_2_ release created an expected pressure build up in the vials, so caution and appropriate glassware is advised. The maximum pressure generated was calculated (Table ST1, Supporting Information) during reaction design to ensure the pressure was well below the 20‐bar rating of the chosen vials.

### 2.3 Scaled Reaction

Three 20 ml crimp top vials containing a magnetic stirrer bar were each filled with PC pellets (1.0 g), acetic acid (30 equiv.), and PPY (0.1 equiv.) The vials were sealed then the headspace purged with nitrogen using a needle through the vial's septum for 2 min. The needles were removed, and the vials placed in an oil bath heated to 180°C and stirred for 3 h. After cooling to room temperature, the vial contents were combined and concentrated to an oil under reduced pressure. The oil was dissolved in methanol (40 ml), followed by addition of 0.5 M KOH (80 ml). The mixture was refluxed for 1 h, cooled to room temperature, and acidified to slight acidity with 4 M HCl added dropwise. The BPA precipitated and was filtered and dried under vacuum. The remaining aqueous solution was extracted with 3 × 50 ml of ethyl acetate and the combined organic layers washed with 50 ml of saturated brine solution and dried under vacuum to yield a yellow solid.

### 2.4 Separation Procedures

Agglomeration method: Following PC/ABS acetolysis (reaction conditions: 0.5 g PC/ABS pellets, 30 molar equivalents of acetic acid, 0.1 molar equivalents of PPY, 180°C, 2 h), 5 ml of ethanol was added while stirring at room temperature, forming a ball of ABS. The ABS ball was rinsed with ethanol and dried in a vacuum oven at 60°C.

Dialysis method: Following PC/ABS acetolysis (reaction conditions: 0.5 g PC/ABS pellets, 30 molar equivalents of acetic acid, 0.1 molar equivalents of PPY, 180°C, 2 h), the reaction mixture was cooled to room temperature then combined with 5 ml of acetone and transferred into a knotted dialysis tube which was subsequently knotted at the open end. The tubing was stirred in 200 ml acetone, with solvent changes at 8 and 16 h. After a total of 32 h the ABS was removed from inside the tubing and dried in a vacuum oven at 60°C. The monomers were recovered by distillation of the acetone fractions under reduced pressure.

### 2.5 Analytical Techniques

Nuclear magnetic resonance (NMR) spectra were run on a Bruker 400 UltraShield or Bruker AVANCE III HD 500 MHz spectrometer at 298 K. Chemical shifts are reported as δ in parts per million (ppm) and referenced to the chemical shift of the residual solvent resonances. The resonance multiplicities are described as s (singlet), d (doublet), or m (multiplet). To quantify monomer yields, 50 μl of crude reaction mixture was added to 600 μl of DMSO‐*d* *6* stock solution containing a known amount of dimethyl sulfone as an internal standard. The integrals for monomer quantification were found using quantitative global spectral deconvolution with four improvement cycles via the peak picking tool in MestReNova version 15.01–35 756. The peak selected for quantification was the dimethyl group which appeared at ^1^H NMR (400 MHz, DMSO‐*d6*) δ: 1.52 (s, 6H), 1.58 (s, 6H), and 1.64 (s, 6H) for BPA, MA‐BPA, and DA‐BPA, respectively. See Supporting Information (Section 1) for more details.

Differential scanning calorimetry (DSC) was performed in triplicate on a DSC 2500 TA instrument under a N_2_ atmosphere using a heat, cool, heat procedure with a starting temperature of −90°C and an ending temperature of 280°C at a heating rate of 10°C/min. Thermogravimetric analysis (TGA) was performed in triplicate on a TA Instruments Discovery SDT 650 under a N_2_ atmosphere using alumina pans. The method consisted of a ramp of 25°C/min to 300°C; an isothermal hold for 15 mins; a high‐resolution ramp of 5°C/min to 600°C (resolution 2, sensitivity 1); an isothermal hold for 10 mins; a ramp of 10°C/min to 800°C; and an isothermal hold for 10.0 mins. Fourier transform infrared spectroscopy (FTIR) was run on a Bruker FTIR Invenio S using 64 scans from 400 to 4000 cm^−1^.

## 3 Results and Discussion

### 3.1 PC Acetolysis

Several PC depolymerization approaches have previously been reported. This work sought to focus on developing a methodology to efficiently and effectively recycle PC/ABS blends and multimaterials. To achieve this, a reaction was sought that could penetrate the PC/ABS blend, facilitating the swift breakdown of the PC component while causing minimal changes to the ABS component. We initially screened the behavior of ABS pellets under methanolysis, glycolysis, and acetolysis conditions to probe how the substrate and reaction conditions interacted (Figure S3, Supporting Information). Glycolysis reactions showed no swelling and led to pellet discoloration, while methanolysis conditions led to only slight pellet swelling. Promisingly, heating in acetic acid (i.e., acetolysis conditions) led the ABS pellets to swell and fragment, forming a milky mixture that suggested acetic acid would help access PC in complex blends. This led us to select acetolysis as the approach to explore further.

**FIGURE 2:**
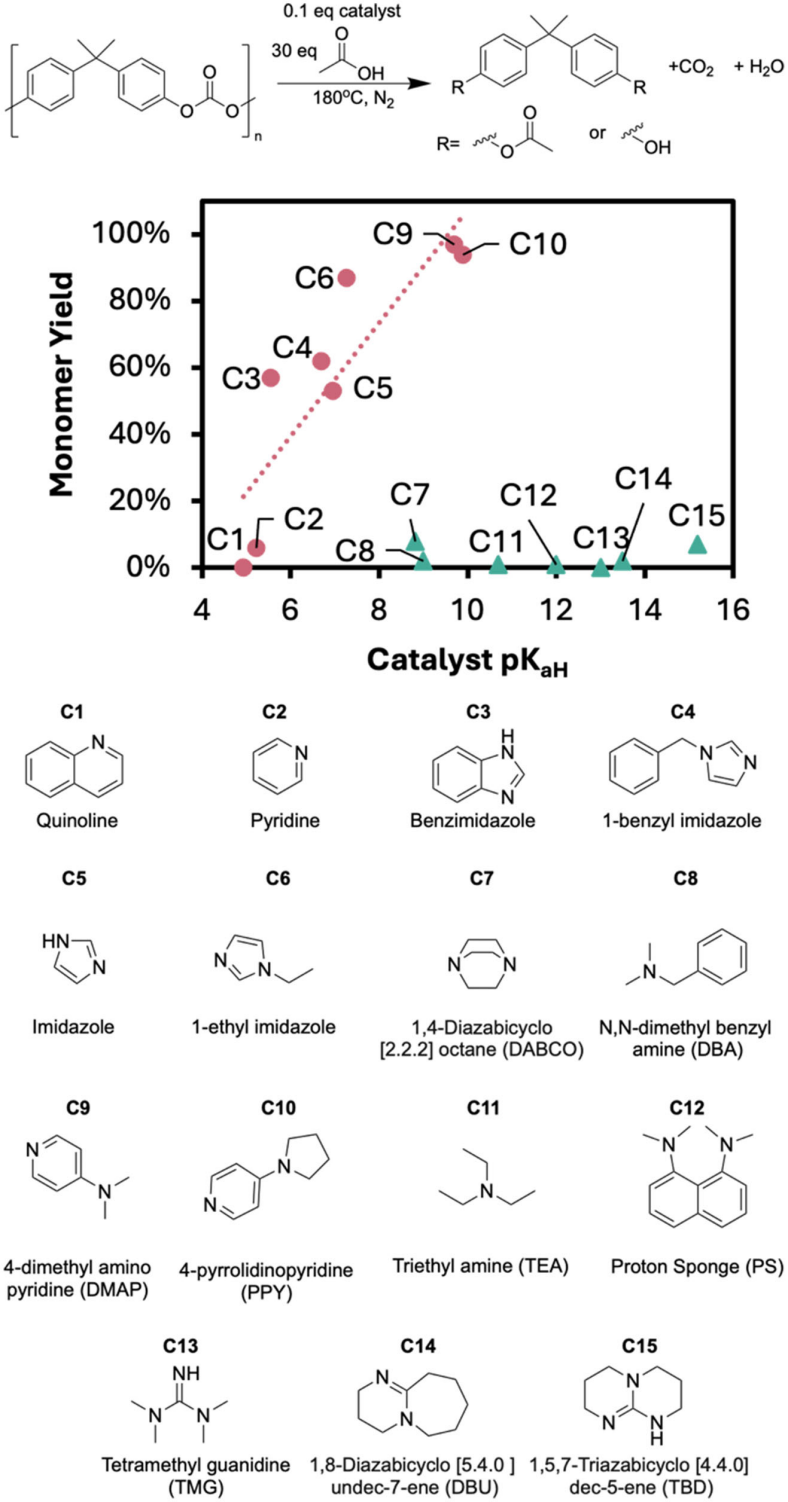
The monomer yield from PC acetolysis for a range of basic organocatalysts (**C1**–**C15**, numbered in order of increasing p*K*_aH_, values were taken from literature and displayed in Table ST3, Supporting Information), showing aromatic nitrogen‐containing catalysts as pink circles (2 h reaction time) and nonaromatic nitrogen‐containing catalysts as green triangles (5‐h reaction time). Reaction conditions: 0.3 g PC, 30 molar equivalents of acetic acid, 0.1 molar equivalents of catalyst, 180°C.

To develop our acetolysis process, we screened 15 organic bases (**C1**–**C15**) using automotive‐grade PC pellets as the challenge substrate (Figure 2). The reactions were performed using 0.3 g PC pellets, 30 molar equivalents of acetic acid and 0.1 equivalents of catalyst (both relative to PC repeat unit), at 180°C. The acetolysis monomer yields, calculated as the sum of mono‐, di‐, and nonacetylated BPA yields, were measured using quantitative ^1^H NMR spectroscopy. In methanolysis and glycolysis reactions, increasing the organocatalyst's basicity increases its effectiveness, leading to “super bases,” such as 1,8‐diazabicyclo [5.4.0]undec‐7‐ene (DBU), predominating [23]. However, this trend was not reproduced in PC acetolysis. A positive correlation between basicity, measured as the p*K*_a_ of the conjugate acid (p*K*_aH_) in water, and the monomer yield from depolymerization at 2 h for catalysts **C1** up to **C10** was observed with two notable outliers, **C8** and **C7**. However, the most basic catalysts, **C11**–**C15** which based on alcoholysis of PET would be expected to be effective, showed extremely limited activity. These inactive catalysts gave less than 10% monomer yield when reaction times were extended to 5 h. The ineffective catalysts included DBU and 1,5,7‐triazabicyclo [4.4.0]dec‐5‐ene, two common transesterification catalysts, which have been demonstrated successfully for PC methanolysis [24, 25]. This highlights the potential differences and considerations for catalyzed acetolysis and the importance of system‐specific catalyst screening. We noted the catalysts that did follow basicity trends all incorporated the basic nitrogen within an aromatic ring, with the most basic catalysts that exhibit this structural feature, **C9**, DMAP, and **C10**, 4‐pyrrolidinyl pyridine (PPY), proving most effective. This realization allowed us to separate the catalysts into the two categories, aromatic nitrogen‐containing catalysts and nonaromatic nitrogen‐containing catalysts, as shown in Figure 2. We hypothesize that the resonance stabilization of the aromatic ring prevents the formation of a tightly bound base‐acetate pair, allowing the catalyst to be active. DMAP and PPY may additionally benefit from their ability to act through either an ordinary basic mechanism (aided by their ability to be both a hydrogen‐bond donor and weak acceptor) or nucleophilic mechanism depending on the reaction [26].

**FIGURE 3:**
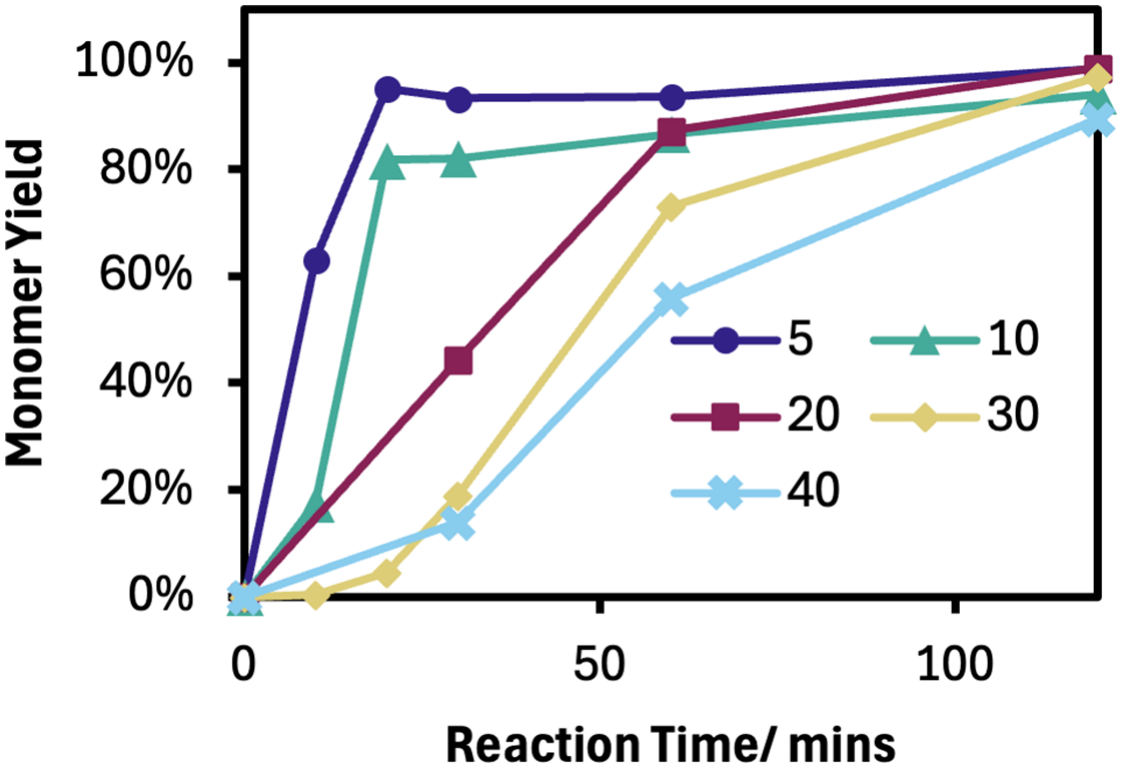
The monomer yield from PC acetolysis with different molar equivalents of acetic acid. Reaction conditions: 0.3 g PC, 0.1 equivalents of DMAP, 180°C. Each point is a separate reaction.

With two catalysts in hand, DMAP was chosen to further optimize the process (Figure S4, S5, Supporting Information). Reducing the temperature to 160°C reduced depolymerization rates with monomer yields reaching 20% after 3 h. Increasing the temperature to 190°C led to a modest increase in rate, with >95% conversion seen at 90 min rather than 120 min. Halving the catalyst loading to 0.05 equivalents significantly slowed down depolymerization (>95% yield in 3 h). Increasing the DMAP loading to 0.2 equivalents offered only modest improvements in reaction times. Interestingly, the equivalents of acetic acid proved an important reaction condition, as reducing from 30 equivalents significantly reduced required reaction times, likely due to the increase in effective catalyst concentrations (Figure 3). In a sorted waste stream of pure PC, lower acetic acid equivalents would facilitate a faster, and likely less energy intensive, recycling process. However, in situations where ABS is present, the larger acetic acid loading is needed to facilitate ABS swelling and the breakdown of the PC within. We therefore maintained the original conditions for mixed material systems.

Under the optimized conditions, there is no significant difference in the catalyst efficacies of DMAP‐ and PPY‐catalyzed PC acetolysis, with both giving near identical yields over eight reaction timepoints (Figure S6, Supporting Information). The health and ecotoxicological hazards of DMAP are significant [27, 28], making the discovery of PPY as an effective catalyst an important step in minimizing reaction hazards (Table ST4, Supporting Information). With safety and applicability considerations in mind, PPY was used to explore the scope of this reaction as a safer alternative catalyst. The quantity of PPY present through PC acetolysis was tracked via ^1^H NMR and did not deviate significantly from 100% suggesting no observable degradation occurred which is a promising feature for potential catalyst recovery and reuse in large scale reactions (Figure S7, Supporting Information).

We performed a scaled‐up reaction consisting of three separate 1 g PC acetolysis reactions which, after 3 h at 180°C were combined. Due to pressure limitations of our reactor vessels scaling up further was not possible (Table ST1, Supporting Information). The acetylated BPA was hydrolyzed and precipitated as fluffy, white crystals which when filtered and dried yielded 1.48 g of BPA (55% yield, >99% purity) (Figure S8, Supporting Information). Extraction of the aqueous solution with ethyl acetate yielded an extra 0.38 g of a sightly yellow solid with a BPA content of 61% therefore further optimization of the purification and separation steps may give higher yields (Figure S9, Supporting Information). Neither fraction of BPA contained detectable traces of PPY catalyst; therefore, catalyst isolation from the aqueous layer could be implemented if catalyst recovery was desirable. The successful isolation of BPA in purities comparable to those required for PC (99.5%) and epoxy (95%) production is an indicator of the feasibility of this process [29].

### 3.2 Acetolysis Kinetics

**FIGURE 4:**
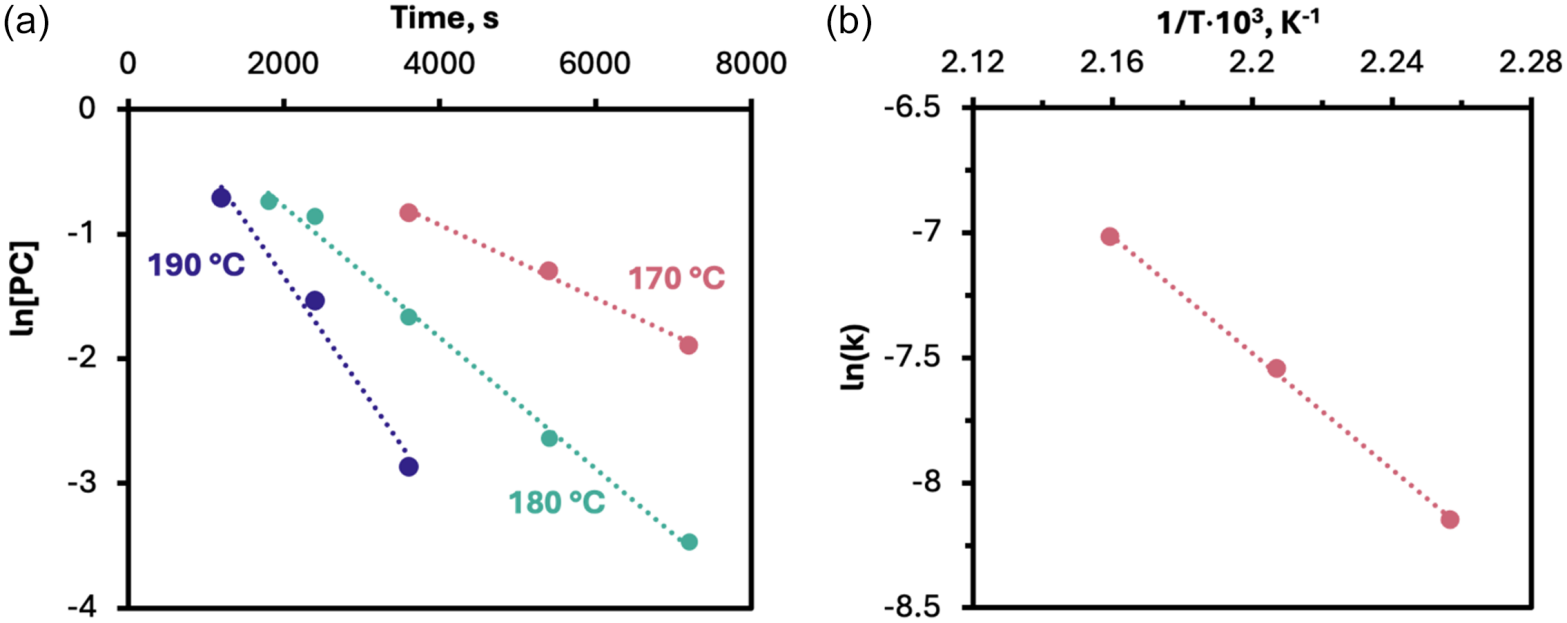
(a) Pseudo first‐order kinetic plots showing the effect of temperature on the rate of PPY‐catalyzed PC acetolysis, and (b) the associated Arrhenius plot for PPY‐catalyzed PC acetolysis. Trend line: *y* = −11.626x + 18.097, *R* ^2^ = 0.9994. Reaction conditions: 0.3 g PC, 0.1 equivalents of PPY, 30 equivalents of acetic acid. [PC] calculated using Equation S3.

Assessing the kinetics and activation energy of this depolymerization explores how energetically demanding the process is, which can aid in facilitating future scale‐up and energy efficiency evaluations. Pseudo first‐order kinetics was viewed as more relevant than alternative kinetic models for depolymerization as an excess of acetic acid permitted the reaction rate to be modeled as proportional to solely the PC concentration [30, 31, 32, 33, 34]. The shrinking particle model [35] does not apply in this instance, as the acetolysis reaction is rapidly homogenized, as the PC begins to depolymerize into soluble oligomers. The continuous distribution model [36, 37], while able to capture detailed molecular weight distributions, was not necessary here as monomer yield was the main metric of interest and therefore its computational demands were unjustified. First‐order kinetic graphs were constructed for 170, 180, and 190°C (Figure 4a). All three temperatures showed good linearity (*R* ^2^ > 0.98) after a short induction time, supporting the use of pseudo first‐order kinetics to model PPY‐catalyzed PC acetolysis (Table ST5, Supporting Information). From this, an Arrhenius plot (Figure 4b) gave an activation energy of 96.7 kJ mol^−1^ and a pre‐exponential factor of 18 s^−1^. The activation energy aligns with values from other PC solvolysis methods (87.6–167 kJ mol^−1^) and is significantly lower than the activation energy for producing BPA by PC oxidative thermal decomposition (362 kJ mol^−1^), highlighting acetolysis as an effective and energy efficient method to recover monomers from polycarbonates [32, 34, 37, 38, 39].

### 3.3 PC/ABS Acetolysis

**FIGURE 5:**
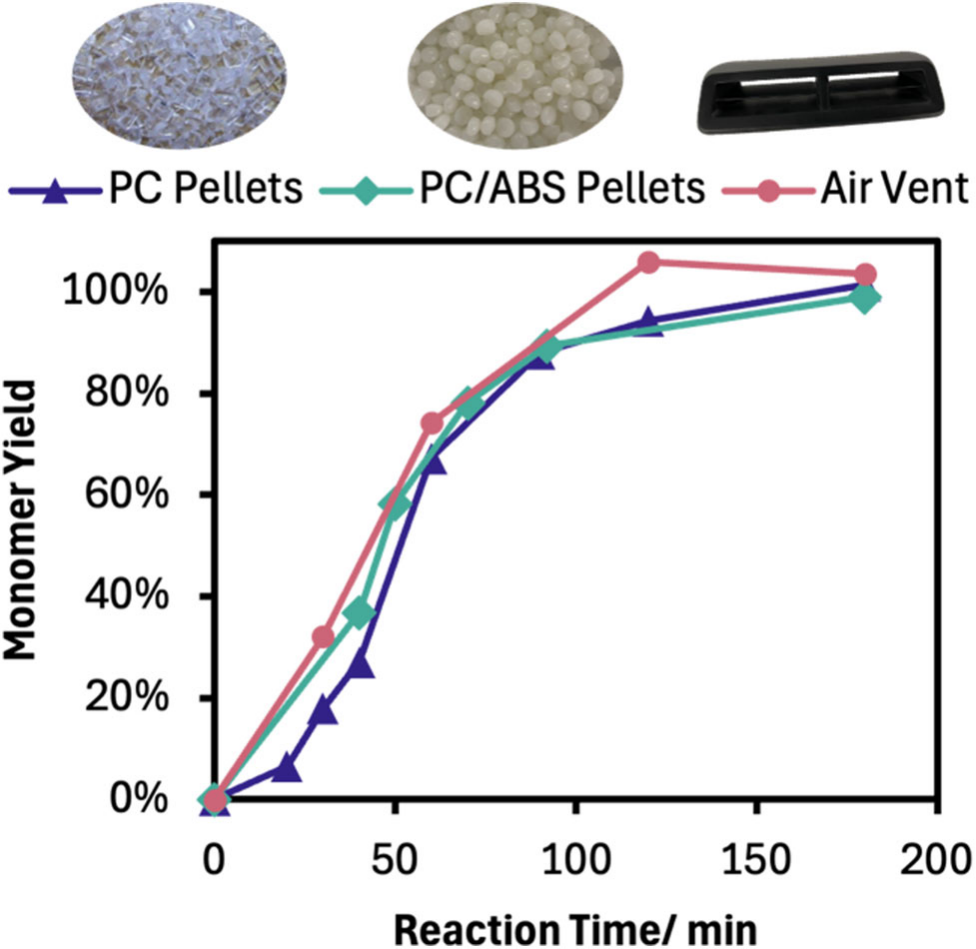
The monomer yield from acetolysis of PC pellets, PC/ABS pellets, and an automotive PC/ABS air vent. Reaction conditions: 0.3 g PC or 0.5 g of PC/ABS pellets or 0.5 g of air vent, 30 equivalents of acetic acid, 0.1 equivalents of PPY, 180°C. Each point is a separate reaction.

With the success of acetolysis on PC pellets, the process was transferred to PC/ABS blends. The commercial resin chosen had 63% PC, determined via NMR spectroscopy (Figure S10, Supporting Information). Encouragingly, the presence of ABS did not hinder the acetolysis of PC, with the progress of the reaction closely matching virgin PC resin (Figure 5). For example, at 90 min the monomer yields for PC and PC/ABS pellets are 88% and 89%, respectively. Though scale‐up was limited due to pressure constraints, a 1.5 g scale reaction led to very similar rates and showed full conversion by 3 h (Figure S11, Supporting Information).

Extending depolymerization methods to a real‐life plastic part is important to assess the robustness of the method beyond model substrates. We tested our acetolysis procedure on an automotive component, namely a PC/ABS‐based air vent used in a Polestar 3. NMR characterization showed the PC content of the air vent was 72% while DSC and FTIR confirmed the material as predominantly PC/ABS (Figure S12–S14, Supporting Information). Depolymerization again followed a near identical pattern to pelletized samples, indeed reaching 100% in a shorter time (120 vs. 180 min), illustrating the recycling process's potential applicability to postconsumer plastic waste.

While these reactions were optimized for BPA recovery from PC/ABS due to its high environmental impact, recovery of usable ABS from this multimaterial should not be discounted. We have previously shown that depolymerization of commercial PET‐G credit cards can be used to recover embedded metals from chip sets and barium from magnetic strips [12, 40], but this feature remains underexplored in chemical recycling of multimaterials. We investigated methods by which a postacetolysis PC/ABS mixture could be separated into a clean ABS fraction and a BPA‐monomers fraction.

Recovery of ABS by simple filtration proved to be impractical due to the small size of the ABS particles (Figure S15). Centrifugation resulted in the ABS phase rising to the top instead of forming a distinct pellet at the bottom making it difficult to separate without the mixing of the two layers (Figure S16). Addition of 5 mL of ethanol postacetolysis, while stirring, helped agglomerate the ABS, which could be filtered and dried in vacuo at 60°C. The recovered ABS accounted for only 85% of the theoretical yield. DSC analysis (Figure S17, Supporting Information) of the sample showed a loss of the PC glass transition (*T*_g_) at 140.4°C indicating effective PC removal while the ABS *T*_g_ remained with no significant change. The virgin PC/ABS pellets also presented a small melting peak at 44.2°C which was not in the recycled material suggesting that part of the ABS, likely a rubber phase, remained in solution rather than agglomerating with the rest of the ABS. Additionally, FTIR confirmed the presence of monomer within the recovered ABS with peaks at 1202 and 1169 cm^−1^ suggesting the presence of acetylated BPA (Figure S18, Supporting Information), therefore alternative separation methods were trialed.

Dialysis, using acetone as the solvent, allowed separation based on the difference in size between the polymeric ABS and monomeric BPA derivatives [41]. In vacuo drying of recovered ABS at 60°C gave improved recovery (93%), the loss of the PC *T*_g_, while also showing no significant shift in ABS *T*_g_, suggesting improved purification of the ABS products (Figure S17, Supporting Information). To further evidence this, TGA showed only a one step degradation compared with a two‐step degradation seen for virgin PC/ABS suggesting complete PC removal (Figure S19, Supporting Information). This improved separation was further supported by FTIR analysis of the collected products (Figure S18, Supporting Information). The purity of the recycled ABS is a hopeful indicator for future mechanical recycling studies beyond the scope of this proof of concept. The small molecule fraction of dialysis was distilled under vacuum to yield an oil with monomer (BPA + MA‐BPA + DA‐BPA) content of 53% translating to 71% monomer yield (Figure S20, Supporting Information). While this suggests some product is lost during the separation stage, further optimization of the dialysis procedure may be able to improve this. Though perhaps not suitable for larger scale processes, dialysis was an effective method to achieve separation of the PC monomers from the ABS. We envision other membrane separation techniques such as ultrafiltration may be more efficient and less resource intensive on an industrial scale [42].

## 4 Conclusion

In conclusion, the depolymerization of PC has been demonstrated using acetic acid to yield acetylated BPA. Base catalyzed acetolysis was investigated and it was discovered that p*K*_a_ was not the defining factor of catalytic activity and there was also a requirement for the basic nitrogen to be within an aromatic ring. Additionally, PPY has been proposed as a safer alternative to DMAP with equivalent activity. Kinetic studies revealed PPY‐catalyzed acetolysis can be modeled using pseudo first‐order kinetics and gave an activation energy of 96.7 kJ mol^−1^ which is in line with other PC depolymerization values. Crucially PC acetolysis works even in blended materials with the presence of ABS not slowing the rate of PC deconstruction in both PC/ABS pellets and an automotive PC/ABS part. This is important due to the current lack of PC/ABS recycling strategies. Postacetolysis, it is possible to separate the PC monomers from the unchanged ABS using a dialysis procedure which enables the whole material to be recycled. The acetylated BPA derivatives were isolated as an oil which could be further purified and processed to yield pure BPA, an energy intensive material to synthesize from fossil feedstocks. The recovered ABS was characterized using DSC, TGA, and FTIR, revealing virgin‐like ABS properties with no detectable trace of PC or PC monomers remaining, enabling the further use of a previously nonreusable material. We aim to further explore the mechanical properties of the recovered ABS in the future, in order to understand how it was affected by the acetolysis procedure and its potential applications within the automotive sector and multisector uses in electronics, toys, and construction.

We have successfully recovered both material components of a complex engineering plastic blend. By this process, we may enable automotive and electronic industries to decrease their reliance on fossil fuels by providing a pathway to high performance recycled feedstocks via selective depolymerization.

## Supporting Information

The data that support the findings of this study are available in the supporting material of this article, including full NMR and FTIR spectra, DSC and TGA curves, kinetics data, and reference information (catalyst p*K*_a_ values and mathematical equations). The authors have cited additional references within the Supporting Information [43, 44, 45, 46, 47, 48]. Additional supporting information can be found online in the Supporting Information Section. **Supporting Fig. S1:** Example of assignment of post acetolysis reaction. NMR solvent is DMSO‐*d*6 with dimethyl sulfone as an internal standard. DMAP was used as the acetolysis catalyst and the reaction produced mono‐ di and non‐ acetylated BPA. **Supporting Fig. S2** **:** Requirement to deconvolute monomer from oligomer peaks before quantifying depolymerization yield. **Supporting Fig. S3:** ABS pellets after heating in solvent at reaction temperature. **Supporting Fig. S4:** The effect of reaction temperature on the monomer yield of PC acetolysis. Reaction conditions: 0.3 g PC, 30 molar equivalents of acetic acid, 0.1 molar equivalents of DMAP. Each point is a separate reaction. **Supporting Fig. S5:** The effect of DMAP catalyst equivalents on the monomer yield of PC acetolysis. Reaction conditions: 0.3 g PC, 30 molar equivalents of acetic acid, 180°C. Each point is a separate reaction. **Supporting Fig. S6:** Monomer yield over time for DMAP and PPY catalysts. Reaction conditions: 0.3 g PC, 30 molar equivalents of acetic acid, 0.1 molar equivalents of catalyst, 180°C. Each point is a separate reaction. **Supporting Fig. S7:** Monitoring of PPY catalyst during reaction. Measured as moles of PPY in reaction at time point by NMR over the moles of PPY weighed in before reaction started. Reaction conditions: 0.3 g PC, 30 molar equivalents of acetic acid, 0.1 molar equivalents of PPY, 180°C. Each point is a separate reaction. **Supporting Fig. S8:** Image and NMR of isolated BPA purity >99% using 1,3,5 trimethoxy benzene as an internal standard. **Supporting Fig. S9:** Image and NMR of isolated BPA purity 61% using 1,3,5 trimethoxy benzene as an internal standard. **Supporting Fig. S10:** Quantification of PC content of PC/ABS pellet. A dried PC/ABS pellet was dissolved in CDCl_3_ solution containing a known amount of 1,3,5 trimethoxy benzene as an internal standard and analyzed via NMR. This was repeated 6 times and gave a mean PC content of 62.5% ± 1.7%. **Supporting Fig. S11:** PC monomer yield over time for PC/ABS pellet acetolysis at 0.5 g and 1.5 g scale. Reaction conditions: 30 molar equivalents of acetic acid, 0.1 molar equivalents of PPY, 180°C. Each point is a separate reaction. **Supporting Fig. S12:** Quantification of PC content of PC/ABS air vent. A dried piece of air vent was dissolved in CDCl_3_ solution containing a known amount of 1,3,5 trimethoxy benzene as an internal standard and analyzed via NMR. This was repeated 6 times and gave a mean PC content of 72% ± 1.5%. **Supporting Fig. S13:** DSC of PC/ABS pellets and PC/ABS air vent. **Supporting Fig. S14:** FTIR of PC/ABS air vent. **Supporting Fig. S15:** Result of filtering post‐acetolysis PC/ABS mixture. **Supporting Fig. S16:** Sample A: post acetolysis PC/ABS was diluted with 7 ml of acetic acid. Sample B: post acetolysis PC/ABS sample was diluted with 6 ml of acetic acid and 1 ml of water. The tubes were spun at 10000 rpm for 45 min at room temperature. **Supporting Fig. S17:** DSC results of PC/ABS pellets and the recycled ABS recovered using dialysis and agglomeration. **Supporting Fig. S18:** FTIR spectra comparing virgin PC/ABS and ABS to post acetolysis recycled ABS. **Supporting Fig. S19:** TGA data of a) ABS separated using dialysis compared to b) virgin PC/ABS. **Supporting Fig. S20:** Oil containing monomers isolated using dialysis quantified using a known amount of dimethyl sulfone in DMSO‐*d*6. **Supporting Table S1:** Pressure inside microwave vials calculations, temperature is 180°C. **Supporting Table S2:** Example calculation for quantifying BPA yield using NMR. The total depolymerization yield is quantified as the sum of the yields of BPA, MA‐BPA and DA‐BPA. **Supporting Table S3:** p*K*_aH_ values for organocatalysts used in the catalyst screen. **Supporting Table S4:** The safety data sheet information for DMAP and PPY.^[27,28]^ **Supporting Table S5:** The linear correlative coefficient (R^2^) value for the three temperatures and the rate constants calculated from the pseudo first‐order kinetic plot seen in Figure 3a.

## Funding

This study was supported by Royal Academy of Engineering (Grant RCSRF2324‐17‐43), Polestar (Grant RCSRF2324‐17‐43), European Regional Development Fund (Grant OC15R19P), and Engineering and Physical Sciences Research Council (Grant EP/R00661X/1, EP/P025021/1 and EP/P025498/1).

## Conflicts of Interest

The authors declare no conflicts of interest.

## Supporting information

Supplementary Material

## Data Availability

The data that support the findings of this study are available in the supplementary material of this article.

## References

[1] T. Maury , N. Tazi , C. T. De Matos , et al., Towards Recycled Plastic Content Targets in New Passenger Cars and Light Commercial Vehicles (Publications Office of The European Union, 2023).

[2] B. Baldassarre , T. Maury , N. Tazi , F. Mathieux , and S. Sala , “Increasing Plastic Circularity in the Automotive Sector: Supply Chain Analysis and Policy Options from the European Union (EU) Resources,” Conservation and Recycling 218 (2025): 108216.

[3] M. Ravina , I. Bianco , B. Ruffino , M. Minardi , D. Panepinto , and M. Zanetti , “Hard‐to‐Recycle Plastics in the Automotive Sector: Economic, Environmental and Technical Analyses of Possible Actions,” Journal of Cleaner Production 394 (2023): 136227.

[4] H. Abedsoltan , “Applications of Plastics in the Automotive Industry: Current Trends and Future Perspectives,” Polymer Engineering & Science 64 (2024): 929–950.

[5] S. S. Soomro , C. Hong , and M. P. Shaver , “Quantification of Recycled Content in Plastics: A Review,” Resources, Conservation and Recycling 221 (2025): 108426.

[6] X. Zhou , Y. Zhai , K. Ren , et al., “Life Cycle Assessment of Polycarbonate Production: Proposed Optimization toward Sustainability,” Resources, Conservation and Recycling 189 (2023): 106765.

[7] E. Orzan , R. Janewithayapun , R. Gutkin , G. Lo Re , and K. Kallio , “Thermo‐Mechanical Variability of Post‐Industrial and Post‐Consumer Recyclate PC‐ABS,” Polymer Testing 99 (2021): 107216.

[8] H.‐T. Chiu , J.‐K. Huang , M.‐T. Kuo , and J.‐H. Huang , “Characterisation of PC/ABS Blend during 20 reprocessing Cycles and Subsequent Functionality Recovery by Virgin Additives,” Journal of Polymer Research 25 (2018): 124.

[9] J. N. Hahladakis , E. Iacovidou , and S. Gerassimidou , “An Overview of the Occurrence, Fate, and Human Risks of the Bisphenol‐A Present in Plastic Materials, Components, and Products,” Integrated Environmental Assessment and Management 19 (2023): 45–62. 35362236 10.1002/ieam.4611

[10] R. A. Clark and M. P. Shaver , “Depolymerization Within a Circular Plastics System,” Chemical Reviews 124 (2024): 2617–2650. 38386877 10.1021/acs.chemrev.3c00739 PMC10941197

[11] C. W. Lahive , S. H. Dempsey , S. E. Reiber , et al., “Acetolysis for Epoxy‐Amine Carbon Fibre‐Reinforced Polymer Recycling,” Nature 642 (2025): 605–612. 40468077 10.1038/s41586-025-09067-y

[12] P. Huang , J. Pitcher , A. Mushing , F. Lourenço , and M. P. Shaver , “Chemical Recycling of Multi‐Materials from Glycol‐Modified Poly(ethylene Terephthalate,” Resources, Conservation and Recycling 190 (2023): 106854.

[13] S. Ügdüler , K. M. V. Geem , R. Denolf , et al., “Towards Closed‐Loop Recycling of Multilayer and Coloured PET Plastic Waste by Alkaline Hydrolysis,” Green Chemistry 22 (2020): 5376–5394.

[14] J. G. Kim , “Chemical Recycling of Poly(bisphenol A Carbonate,” Polymer Chemistry 11 (2020): 4830–4849.

[15] P.‐H. Rathsack , D. Scheithauer , J. Kleeberg , and M. Gräbner , “Chemical Recycling of PC/ABS‐Blends by Pyrolysis,” Journal of Analytical and Applied Pyrolysis 188 (2025): 107047.

[16] M. Li , W. Wang , and J. Yu , “Comparison of the Pyrolysis Behavior of PC, ABS and PC/ABS,” Journal of Analytical and Applied Pyrolysis 183 (2024): 106774.

[17] E. C. Vouvoudi , A. T. Rousi , and D. S. Achilias , “Effect of the Catalyst Type on Pyrolysis Products Distribution of Polymer Blends Simulating Plastics Contained in Waste Electric and Electronic Equipment,” Sustainable Chemistry and Pharmacy 34 (2023): 101145.

[18] D. Parida , A. Aerts , L. Vargas Perez , et al., “Boosting Methanolysis of Polycarbonate (PC) for Bisphenol A Recovery from End‐of‐Life PC and PC/ABS Blend,” Chemical Engineering Journal 497 (2024): 154390.

[19] M. Comí , M. Thys , A. Aerts , et al., “Revealing the Dynamics of Sustainable Epoxy‐Acrylate Networks from Recycled Plastics Blends and Oligomeric Lignin Precursors,” ChemSusChem 18 (2025): e202402375. 39801250 10.1002/cssc.202402375

[20] C. Marquez , A. Aerts , D. Parida , et al., “Monomer Recycling of Virgin Polycarbonate (PC), End‐of‐Life PC and PC‐ABS Blends by Ni‐Catalyzed Reductive Depolymerization,” Green Chemistry 27 (2025): 5709, 10.1039/D4GC06438K.

[21] Y. Peng , J. Yang , C. Deng , J. Deng , L. Shen , and Y. Fu , “Acetolysis of Waste Polyethylene Terephthalate for Upcycling and Life‐Cycle Assessment Study,” Nature Communications 14 (2023): 3249. 10.1038/s41467-023-38998-1 PMC10241940 37277365

[22] C. Alberti , F. Scheliga , and S. Enthaler , “Depolymerization of End‐of‐Life Poly(bisphenol A Carbonate) via Transesterification with Acetic Anhydride as Depolymerization Reagent,” ChemistrySelect 4 (2019): 2639–2643.

[23] C. Jehanno , M. M. Pérez‐Madrigal , J. Demarteau , H. Sardon , and A. P. Dove , “Organocatalysis for Depolymerisation,” Polymer Chemistry 10 (2018): 172–186.

[24] E. Quaranta , D. Sgherza , and G. Tartaro , “Depolymerization of Poly(bisphenol A Carbonate) under Mild Conditions by Solvent‐Free Alcoholysis Catalyzed by 1,8‐Diazabicyclo[5.4.0]undec‐7‐ene as a Recyclable Organocatalyst: A Route to Chemical Recycling of Waste Polycarbonate,” Green Chemistry 19 (2017): 5422–5434.

[25] T. Do , E. R. Baral , and J. G. Kim , “Chemical Recycling of Poly(bisphenol A Carbonate): 1,5,7‐Triazabicyclo[4.4.0]‐Dec‐5‐ene Catalyzed Alcoholysis for Highly Efficient Bisphenol A and Organic Carbonate Recovery,” Polymer 143 (2018): 106–114.

[26] E. Larionov and H. Zipse , “Organocatalysis: Acylation Catalysts,” WIREs Computational Molecular Science 1 (2011): 601–619.

[27] Sigma Aldrich , “4‐(Dimethylamino)pyridine Safety Data Sheet,” 2024, accessed June 14, 2025, https://www.sigmaaldrich.com/GB/en/product/aldrich/107700?srsltid=AfmBOor5Z7JTaSOetgDsHrmYUFtcUjC93m7Xg0Q7nIj6Hn7XEu9KcijA.

[28] Sigma Aldrich , “4‐Pyrrolidinopyridine Safety Data Sheet,” 2024, accessed June 14, 2025, https://www.sigmaaldrich.com/GB/en/product/aldrich/213373?srsltid=AfmBOoqAASD2mAYBVzXx_C5pE9xgEvuRO_0_aZysKq‐XCwdqL1WJiP8b.

[29] G. M. Kissinger and R. Sato , Process for the Manufacture of Bisphenol‐A, USH1943H1, 2001.

[30] R.‐J. L. Peterson , E. P. Neppel , D. Holmes , P. A. Trinh , R. Y. Ofoli , and J. R. Dorgan , “Upcycling of Waste Poly(ethylene Terephthalate): Ammonolysis Kinetics of Model Bis(2‐Hydroxyethyl Terephthalate) and Particle Size Effects in Polymeric Substrates,” ChemSusChem 18 (2025): e202500509. 40437697 10.1002/cssc.202500509 PMC12302304

[31] G. A. Morris , J. Castile , A. Smith , G. G. Adams , and S. E. Harding , “The Kinetics of Chitosan Depolymerisation at Different Temperatures,” Polymer Degradation and Stability 94 (2009): 1344–1348.

[32] F. Liu , L. Li , S. Yu , Z. Lv , and X. Ge , “Methanolysis of Polycarbonate Catalysed by Ionic Liquid [Bmim][Ac],” Journal of Hazardous Materials 189 (2011): 249–254. 21402441 10.1016/j.jhazmat.2011.02.032

[33] H. Wang , Z. Li , Y. Liu , X. Zhang , and S. Zhang , “Degradation of Poly(ethylene Terephthalate) Using Ionic Liquids,” Green Chemistry 11 (2009): 1568–1575.

[34] L.‐C. Hu , A. Oku , and E. Yamada , “Alkali‐Catalyzed Methanolysis of Polycarbonate. A Study on Recycling of Bisphenol A and Dimethyl Carbonate,” Polymer 39 (1998): 3841–3845.

[35] H. Torabi , F. Javi , T. W. Deisenroth , T. V. Pho , V. Barbright , and A. Abbaspourrad , “Mechanism and Kinetics of Enzymatic Degradation of Polyester Microparticles Using a Shrinking Particle–shrinking Core Model,” Lab on a Chip 23 (2023): 4456–4465. 37740368 10.1039/d3lc00581j

[36] D. Kim , B. Kim , Y. Cho , M. Han , and B.‐S. Kim , “Kinetics of Polycarbonate Glycolysis in Ethylene Glycol,” Industrial & Engineering Chemistry Research 48 (2009): 685–691.

[37] H. Jie , H. Ke , Z. Qing , C. Lei , W. Yongqiang , and Z. Zibin , “Study on Depolymerization of Polycarbonate in Supercritical Ethanol,” Polymer Degradation and Stability 91 (2006): 2307–2314.

[38] R. Piñero‐Hernanz , J. García‐Serna , and M. J. Cocero , “Nonstationary Model of the Semicontinuous Depolymerization of Polycarbonate,” AIChE Journal 52 (2006): 4186–4199.

[39] Y. Kitahara , S. Takahashi , M. Tsukagoshi , and T. Fujii , “Formation of Bisphenol A by Thermal Degradation of Poly(bisphenol A Carbonate),” Chemosphere 80 (2010): 1281–1284. 20630563 10.1016/j.chemosphere.2010.06.053

[40] P. Huang , A. Ahamed , R. Sun , et al., “Circularizing PET‐G Multimaterials: Life Cycle Assessment and Techno‐Economic Analysis,” ACS Sustainable Chemistry and Engineering 11 (2023): 15328–15337. 37886038 10.1021/acssuschemeng.3c04047 PMC10598876

[41] T. Schuett , I. Anufriev , P. Endres , et al., “A User‐Guide for Polymer Purification Using Dialysis,” Polymer Chemistry 14 (2022): 92–101.

[42] H. Strathmann , Ullmann's Encyclopedia of Industrial Chemistry 22 (John Wiley & Sons, Ltd., 2011): 413–456.

[43] Y. Zhang , C. Peng , G. Wang , X. Huang , and Y. Yu , “Hydroxyl‐Free Epoxy Thermoplastics from Active Esters with Low Dielectric Constant and Water Sorption,” Acs Applied Electronic Materials 6 (2024): 4578–4586.

[44] S. Tshepelevitsh , A. Kütt , M. Lõkov , et al., “On the Basicity of Organic Bases in Different Media,” European Journal of Organic Chemistry 2019 (2019): 6735–6748.

[45] C. Mao , Z. Wang , P. Ji , and J.‐P. Cheng , “Is Amine a Stronger Base in Ionic Liquid Than in Common Molecular Solvent? An Accurate Basicity Scale of Amines,” The Journal of Organic Chemistry 80 (2015): 8384–8389. 26218631 10.1021/acs.joc.5b01200

[46] B. Lenarcik and P. Ojczenasz , “The Influence of the Size and Position of the Alkyl Groups in Alkylimidazole Molecules on Their Acid‐Base Properties,” Journal of Heterocyclic Chemistry 39 (2002): 287–290.

[47] E. Marzocchi , S. Grilli , L. Della Ciana , L. Prodi , M. Mirasoli , and A. Roda , “Chemiluminescent Detection Systems of Horseradish Peroxidase Employing Nucleophilic Acylation Catalysts,” Analytical Biochemistry 377 (2008): 189–194. 18394418 10.1016/j.ab.2008.03.020

[48] A. Toulmin , J. M. Wood , and P. W. Kenny , “Toward Prediction of Alkane/Water Partition Coefficients,” Journal of Medicinal Chemistry 51 (2008): 3720–3730. 18558667 10.1021/jm701549s

