# Supplementary material for: Chemical Recycling of Polycarbonate Acrylonitrile Butadiene Styrene Blends via Organocatalyzed Acetolysis
Source: ChemSusChem. 2026 Jan 5;19(1):e202502161. doi: 10.1002/cssc.202502161 (PMC12766866; doi:10.1002/cssc.202502161)
Supplement: Supplementary file 1 — Supplementary Material [file CSSC-19-e202502161-s001.pdf]

Chemical Recycling of Polycarbonate Acrylonitrile Butadiene Styrene (PC/ABS) Blends via Organocatalyzed Acetolysis

Supporting Information

Mary E. Pool[a], Edward Savage[a], Rachel Holland[b], Ciaran W. Lahive*[a] and Michael P. Shaver[a]

[a] M. E. Pool, E. Savage, Dr. C. W. Lahive, Prof. M. P. Shaver

Department of Materials, The University of Manchester, Manchester, M1 7DN, UK

E-mail: ciaran.lahive@manchester.ac.uk, michael.shaver@manchester.ac.uk

[b] Dr R. Holland

Polestar Automotive UK Ltd, Envisage, Unit F, Progress Close, Binley, Coventry, CV3 2TF

Contents

Table ST1. Pressure calculations Page 3

Section 1 NMR of products and yield calculations
NMR Spectra of monomers for assignment Page 3
Figure S1. Example of assignment of post acetolysis reaction Page 4
Figure S2. Deconvolution of monomer peaks. Page 5
Equation SE1. Monomer quantification from NMR Page 5
Equation SE2. Total monomer yield calculation Page 5
Table ST1. Example calculation for quantifying BPA yield using NMR Page 5

Section 2 PC acetolysis
Figure S3. ABS pellets after heating in solvent at reaction temperature Page 6
Table ST2. pK_{aH} values for organocatalysts Page 6
Figure S4. The effect of reaction temperature on the monomer yield Page 7
Figure S5. The effect of DMAP loading on the monomer yield Page 7
Figure S6. Monomer yield over time for DMAP and PPY catalysts Page 7
Table ST4. The safety data sheet information for DMAP and PPY Page 8
Figure S7. Monitoring of PPY catalyst during reaction Page 8
Figure S8. Image and NMR of isolated BPA purity >99% Page 9
Figure S9. Image and NMR of isolated BPA purity 61% Page 9

Section 3 PC acetolysis kinetics
Equation SE3. Determining the concentration of PC Page 9
Table S2. Pseudo first-order kinetic plot data Page 9

Section 4 PC/ABS Acetolysis
Figure S10. Quantification of PC content of PC/ABS pellet Page 10
Figure S11. PC/ABS pellet acetolysis at 0.5 g and 1.5 g scale Page 10
Figure S12. Quantification of PC content of PC/ABS air vent Page 10
Figure S13. DSC of PC/ABS pellets and PC/ABS based air vent. Page 11
Figure S14. FTIR of PC/ABS based air vent. Page 11
Figure S15. Result of filtering post-acetolysis PC/ABS mixture. Page 11
Figure S16. Result of centrifugation of post-acetolysis PC/ABS mixture. Page 12
Figure S17. DSC results of recycled ABS Page 12
Figure S18. FTIR results of recycled ABS Page 13
Figure S19. TGA results of recycled ABS Page 13
Figure S20. NMR of monomers isolated using dialysis Page 14

References Page 14

Table ST1: Pressure inside microwave vials calculations, temperature is 180°C.

	Volume acetic acid/ ml	Head space / ml	Mass PC/ g	Max CO_2 yield/ mmols	Pressure CO_2/ Bar[a]	Pressure acetic acid/ Bar[b]	Total Pressure / Bar
**0.3 g PC, 30 eq acetic acid**	2.02	18.0	0.30	1.18	2.47	5.11	7.58
**1 g PC, 30 eq acetic acid**	6.75	13.3	1.00	3.93	11.2	5.11	16.3
**0.5 g PC/ABS, 30 eq acetic acid**	2.11	17.9	0.31	1.22	2.57	5.11	7.68
**1.5 g PC/ABS, 30 eq acetic acid**	6.37	13.6	0.93	3.66	10.1	5.11	15.2

[a] calculated using the ideal gas equation, [b] calculated using the Antoine parameters

Section 1: NMR of products and yield calculations

Bisphenol-A (BPA) was purchased from Sigma Aldrich and analyzed using NMR: 1H NMR (400 MHz, DMSO-*d6*) δ 9.21 (s, 2H, d), 6.97 (m, 4H, b), 6.63 (m, 4H, c), 1.52 (s, 6H, a).

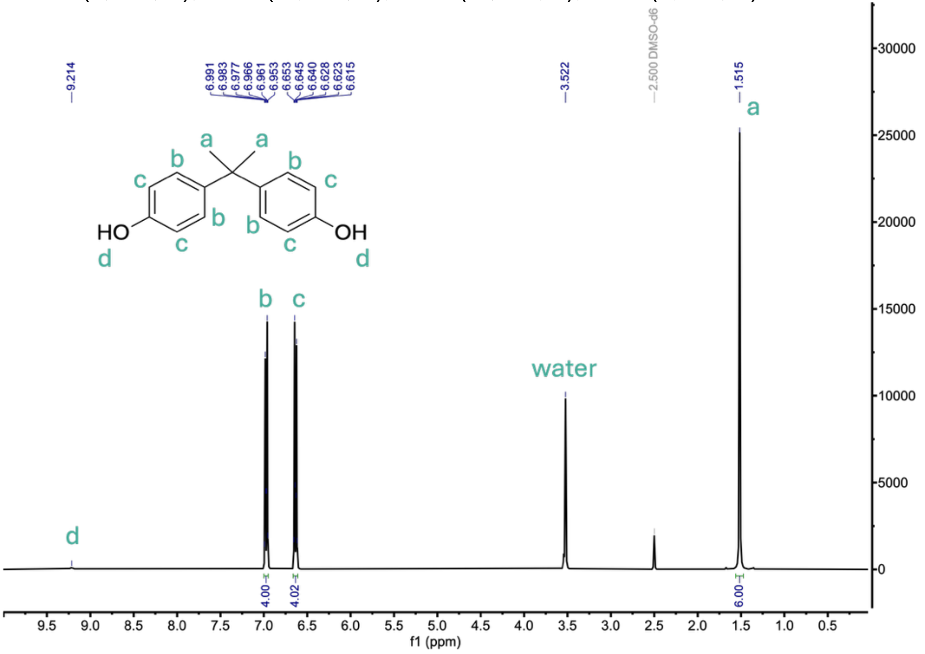

Diacetylated BPA (DA-BPA) was synthesized using a method from Zang et al.[44] and analyzed using NMR: ^{1}H NMR (400 MHz, DMSO-*d6*) δ 7.25 (m, 4H, b), 7.03 (m, 4H, c), 2.24 (s, 6H, d), 1.64 (s, 6H, a).

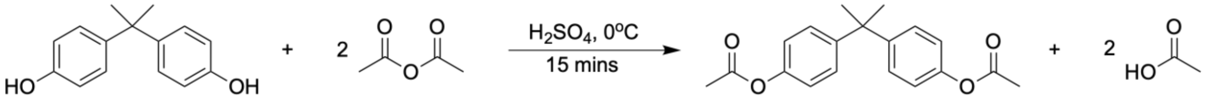

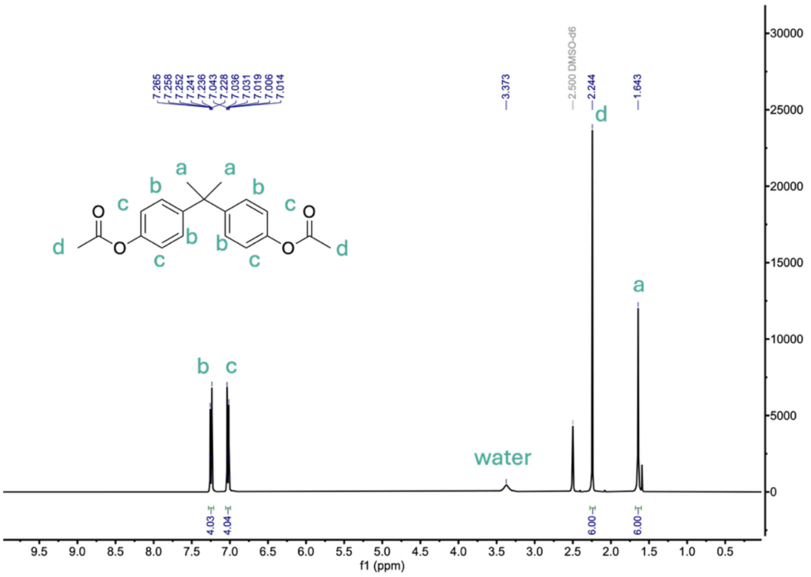

By elimination of the peaks given by BPA and DA-BPA, the peaks for mono-acetylated BPA (MA-BPA) can be assigned from a crude post acetolysis reaction (see **Figure S1**) as follows: ^{1}H NMR (400 MHz, DMSO-*d6*) δ 9.19 (br s, 1H, a), 7.21 (m, 2 H, b), 7.01 (m, 2 H, c), 6.99 (m, 2 H, d), 6.66 (m, 2H, e), 2.24 (s, 3H, f), 1.58 (s, 6H, g).

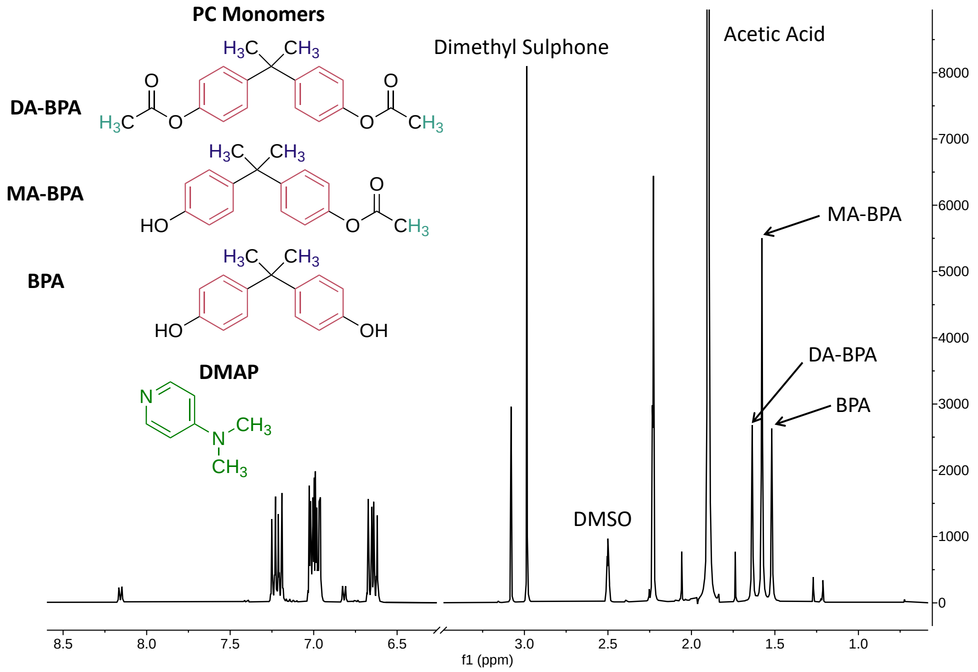

Figure S1. Example of assignment of post acetolysis reaction. NMR solvent is DMSO-d6 with dimethyl sulfone as an internal standard. DMAP was used as the acetolysis catalyst and the reaction produced mono- di and non- acetylated BPA.

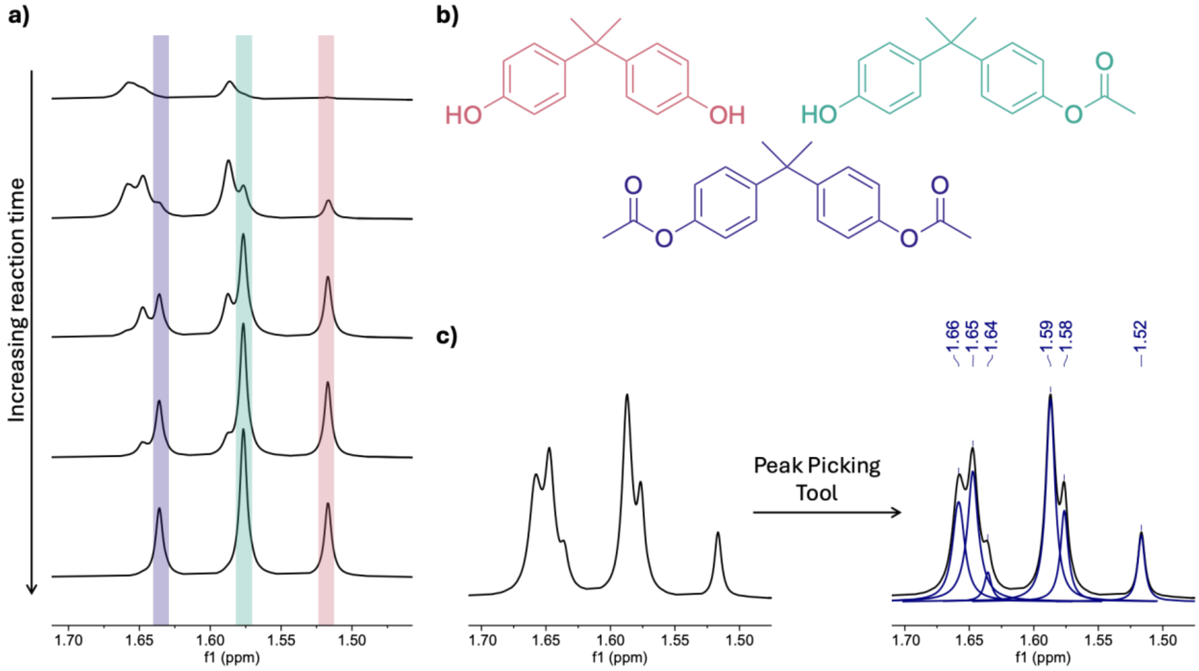

Figure S2. Requirement to deconvolute monomer from oligomer peaks before quantifying depolymerization yield.

Equation SE1. Calculating the moles of BPA produced in acetolysis where n is moles, I is the integration of the NMR signal and N is the number of protons contributing to that signal. Here, dimethyl sulfone ($DMSO_2$) is used as the internal standard. This equation can be adapted to calculate the mols of MA-BPA and DA-BPA which appear at different chemical shifts.

$$n_{BPA} = \frac{I_{BPA}}{I_{DMSO_2}} \times \frac{N_{DMSO_2}}{N_{BPA}} \times n_{DMSO_2}$$

Equation SE2. Calculating the percentage yield of a PC acetolysis reaction using the sum of monomer moles and the original mols of PC repeating units.

$$Monomer\ yield = \frac{n_{BPA} + n_{DA\_BPA} + n_{MA\_BPA}}{n_{PC}} \times 100\%$$

Table ST2. Example calculation for quantifying BPA yield using NMR. The total depolymerization yield is quantified as the sum of the yields of BPA, MA-BPA and DA-BPA.

**PC**	A	Mass in reaction	0.2975	g	
	B	MW of repeating group	254.28	g/mol	
	C	Mols in reaction	1.170	mmol	=A/B
**Acetic acid**	D	Volume in reaction	2.007	ml	
**Stock solution**	E	MW of Standard	94.13	g/mol	
	F	Mass of Standard	0.0235	g	
	G	Volume solvent in stock	9.000	ml	
	H	Concentration of stock	0.0026	g/ml	=F/G
**NMR Sample**	I	Volume of Crude Reaction	0.050	ml	
	J	Volume of Stock Solution	0.700	ml	
	K	Mass of Standard	0.0018	g	=H*J
	L	Mols of Standard	0.0194	mmol	=K/E
**NMR Interpretation**	M	Dimethyl integration	32364		
	N	Dimethyl proton number	6.00		
	O	Standard integration	89203		
	P	Standard proton number	6.00		
**Yield Calculation**	Q	Mols of BPA in sample	0.0070	mmol	=M/O*P/N*L
	R	Mols of BPA in reaction	0.2828	mmol	=D/I*Q
	S	BPA Yield	24.17	%	=R/C*100%

Section 2 PC acetolysis

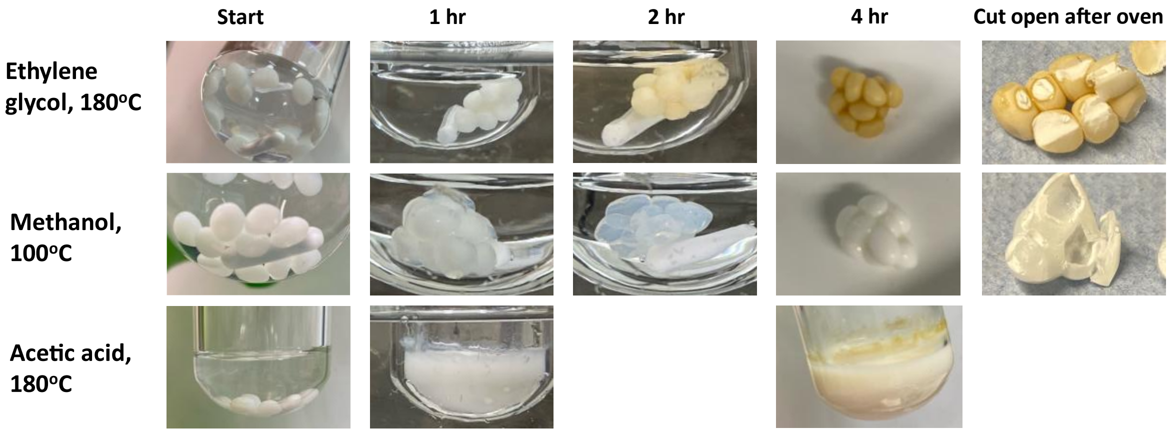

Figure S3. ABS pellets after heating in solvent at reaction temperature.

Table ST3. pK_{aH} values for organocatalysts used in the catalyst screen.

	pK_{aH}	Reference
1,1,3,3-Tetramethylguanidine	13.0	45
1,4-diazabicyclo [2.2. 2]octane	8.82	45
Proton Sponge	12.0	46
Imidazole	6.95	45
1-ethyl imidazole	7.26	47
Pyridine	5.23	45
4-Pyrrolidinylpyridine	9.9	48
4-Dimethylaminopyridine	9.7	48
Benzimidazole	5.56	45
1-Benzyl imidazole	6.7	49
1,8-Diazabicyclo [5.4.0] undec-7-ene	13.5	45
1,5,7-Triazabicyclo [4.4.0] dec-5-ene	15.2	45
Triethylamine	10.7	46
Quinoline	4.93	45
Dimethyl benzyl amine	9.0	46

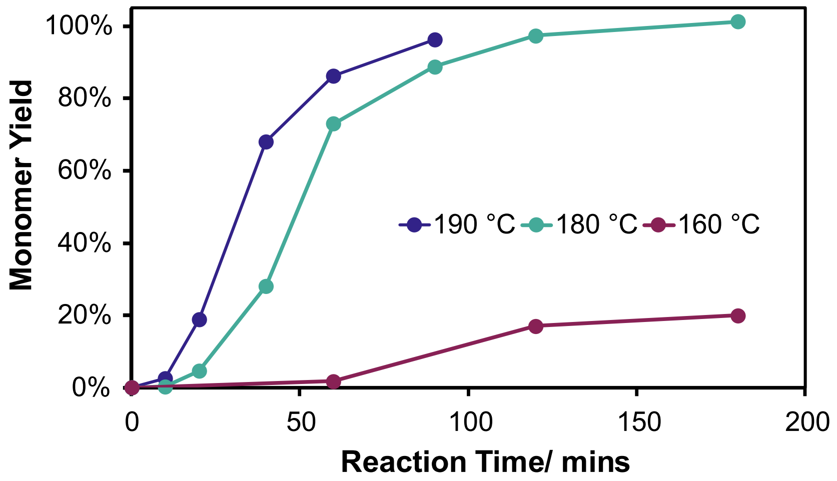

Figure S4. The effect of reaction temperature on the monomer yield of PC acetolysis. Reaction conditions: 0.3 g PC, 30 molar equivalents of acetic acid, 0.1 molar equivalents of DMAP. Each point is a separate reaction.

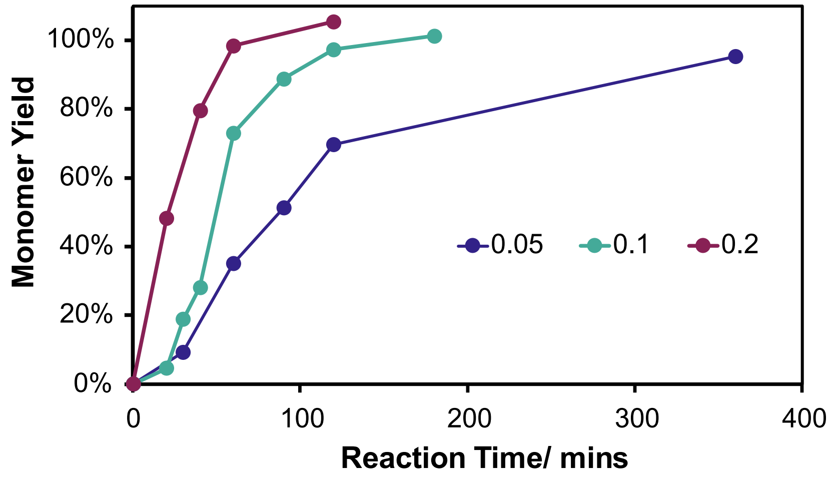

Figure S5. The effect of DMAP catalyst equivalents on the monomer yield of PC acetolysis. Reaction conditions: 0.3 g PC, 30 molar equivalents of acetic acid, 180 °C. Each point is a separate reaction.

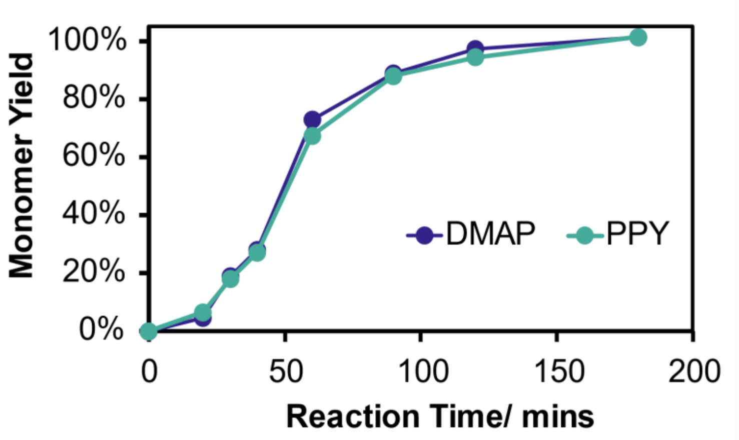

Figure S6. Monomer yield over time for DMAP and PPY catalysts. Reaction conditions: 0.3 g PC, 30 molar equivalents of acetic acid, 0.1 molar equivalents of catalyst, 180 °C. Each point is a separate reaction.

Table ST4. The safety data sheet information for DMAP and PPY.[27,28]

	**DMAP**	**PPY**
**Pictogram**	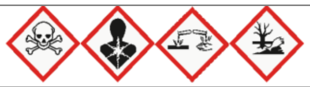	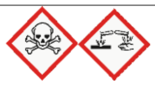
**Signal Word**	Danger	Danger
**Hazard Statements**	H301 + H331 Toxic if swallowed or if inhaled.<br>H310 Fatal in contact with skin.<br>H315 Causes skin irritation.<br>H318 Causes serious eye damage.<br>H370 Causes damage to organs (Nervous system).<br>H411 Toxic to aquatic life with long lasting effects.	H301 Toxic if swallowed. H314 Causes severe skin burns and eye damage.

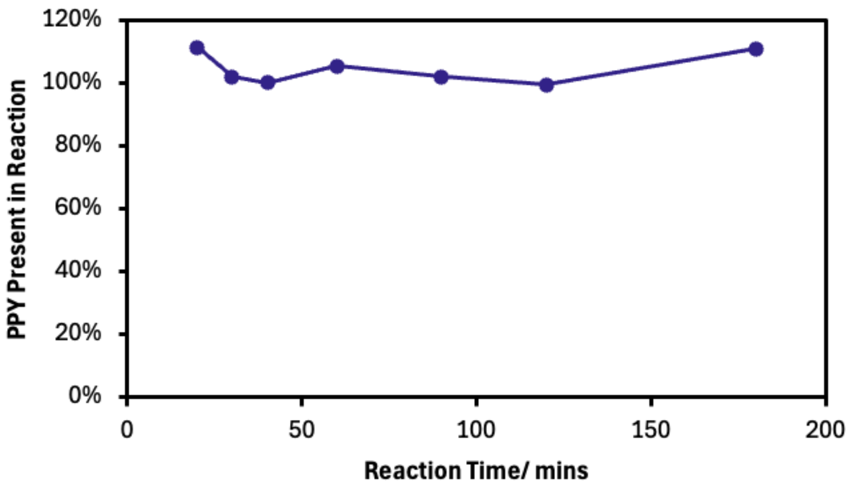

Figure S7. Monitoring of PPY catalyst during reaction. Measured as moles of PPY in reaction at time point by NMR over the moles of PPY weighed in before reaction started. Reaction conditions: 0.3 g PC,

30 molar equivalents of acetic acid, 0.1 molar equivalents of PPY, 180 °C. Each point is a separate reaction.

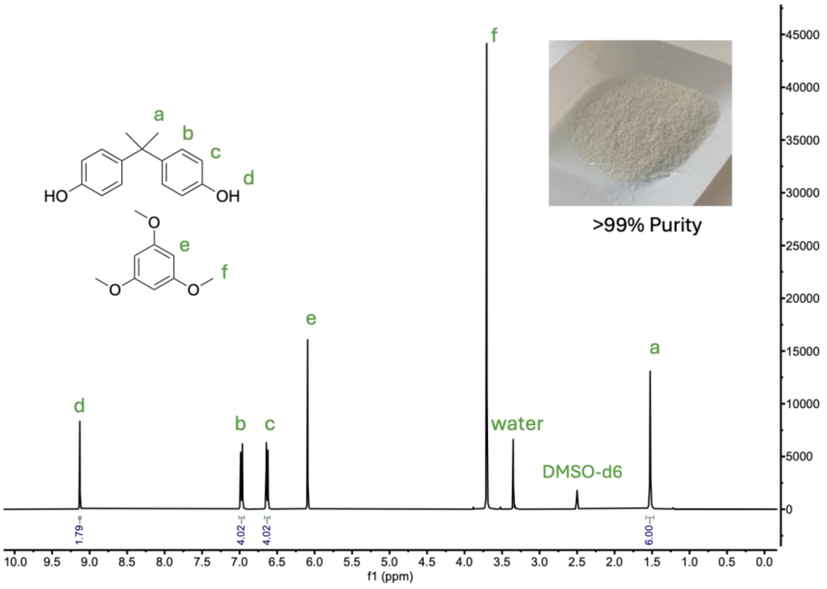

Figure S8. Image and NMR of isolated BPA purity >99% using 1,3,5 trimethoxy benzene as an internal standard

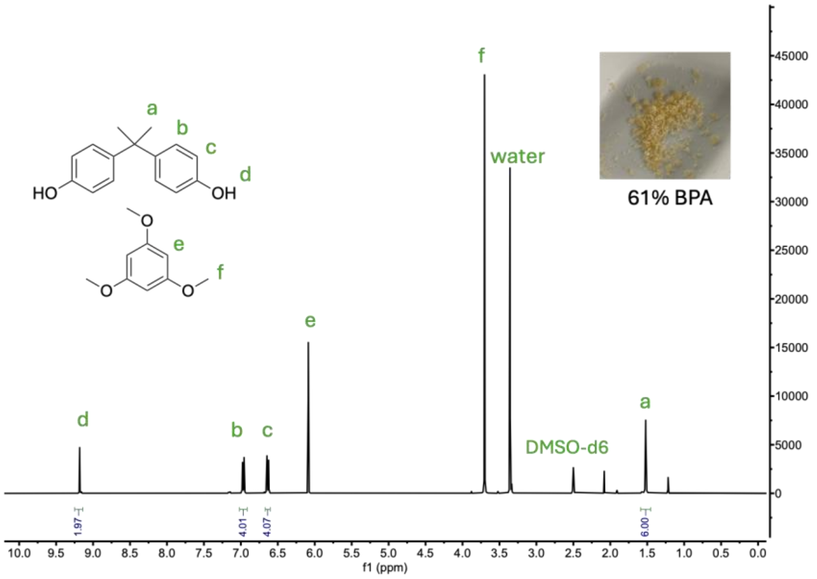

Figure S9. Image and NMR of isolated BPA purity 61% using 1,3,5 trimethoxy benzene as an internal standard

Section 3 PC Acetolysis Kinetics

Equation SE3. Determining the concentration of PC for pseudo first-order kinetics calculations.

$$[PC] = \frac{Mols\ PC\ repeating\ units_{initial} - Mols\ monomers_{reaction\ time}}{Volume\ of\ acetic\ acid}$$

Table ST5. The linear correlative coefficient (R^2) value for the three temperatures and the rate constants calculated from the pseudo first-order kinetic plot seen in **Figure 3a**.

Temperature/ °C	Linear correlative coefficient	Rate Constant/ s^{-1}

**170**	0.995	0.00029
**180**	0.994	0.00053
**190**	0.982	0.00090

Section 4 PC/ABS Acetolysis

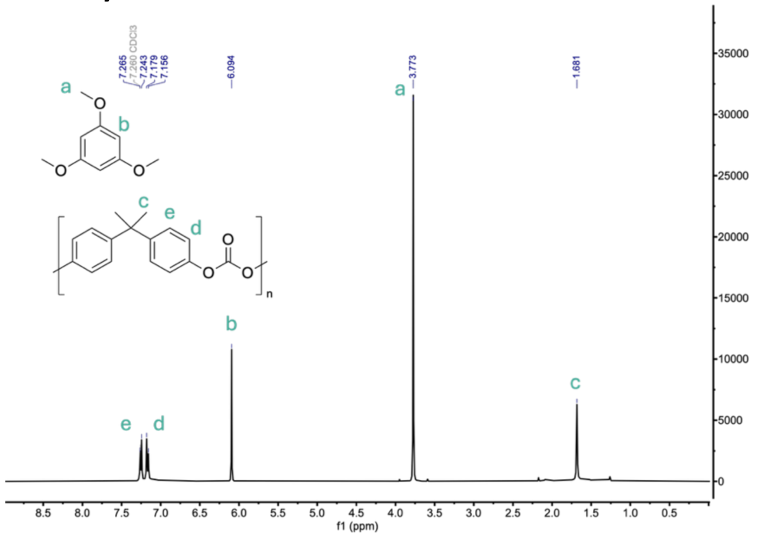

Figure S10. Quantification of PC content of PC/ABS pellet. A dried PC/ABS pellet was dissolved in $CDCl_3$ solution containing a known amount of 1,3,5 trimethoxy benzene as an internal standard and analyzed via NMR. This was repeated 6 times and gave a mean PC content of 62.5% ± 1.7%.

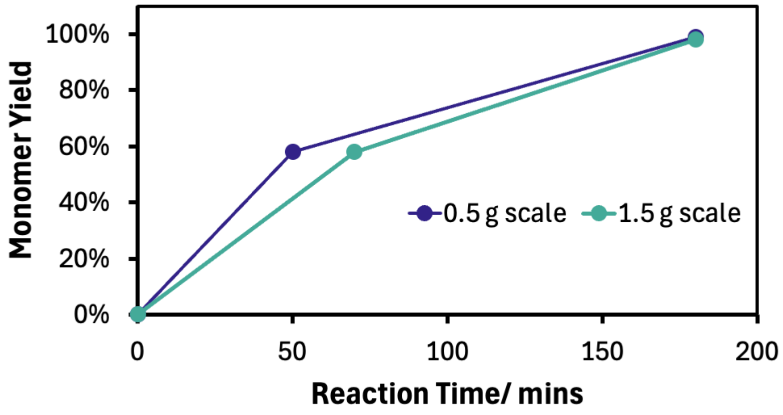

Figure S11. PC monomer yield over time for PC/ABS pellet acetolysis at 0.5 g and 1.5 g scale. Reaction conditions: 30 molar equivalents of acetic acid, 0.1 molar equivalents of PPY, 180 °C. Each point is a separate reaction.

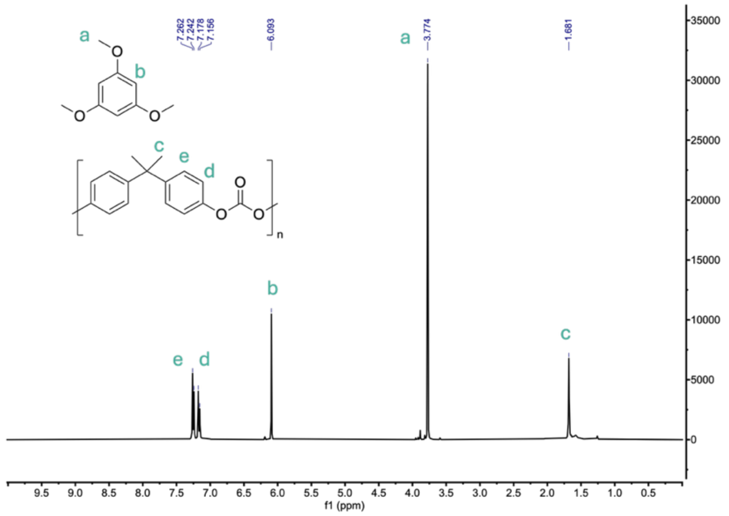

Figure S12. Quantification of PC content of PC/ABS air vent. A dried piece of air vent was dissolved in $CDCl_3$ solution containing a known amount of 1,3,5 trimethoxy benzene as an internal standard and analyzed via NMR. This was repeated 6 times and gave a mean PC content of 72% ± 1.5%.

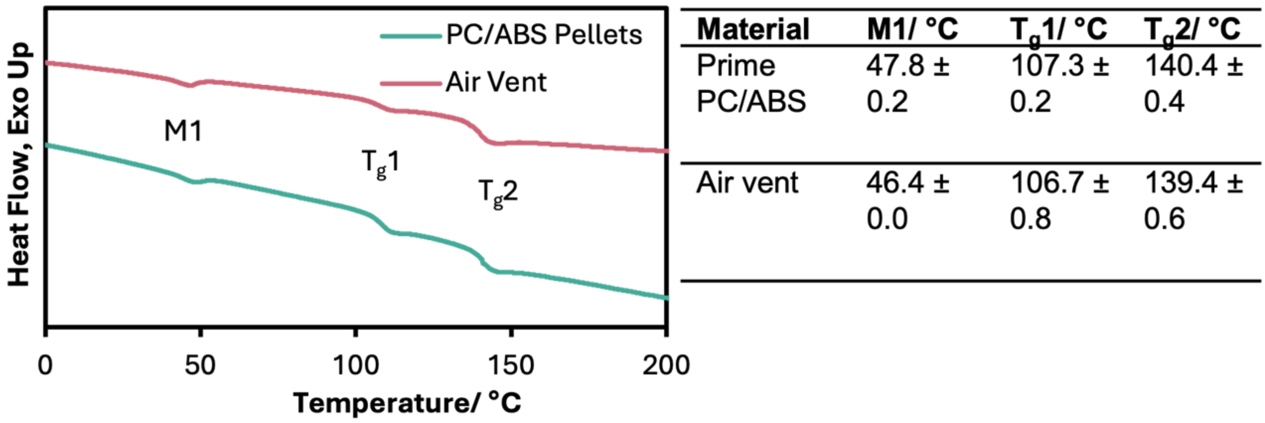

Material	M1/ °C	T_g1/ °C	T_g2/ °C
Prime PC/ABS	47.8 ± 0.2	107.3 ± 0.2	140.4 ± 0.4
Air vent	46.4 ± 0.0	106.7 ± 0.8	139.4 ± 0.6

Figure S13. DSC of PC/ABS pellets and PC/ABS air vent.

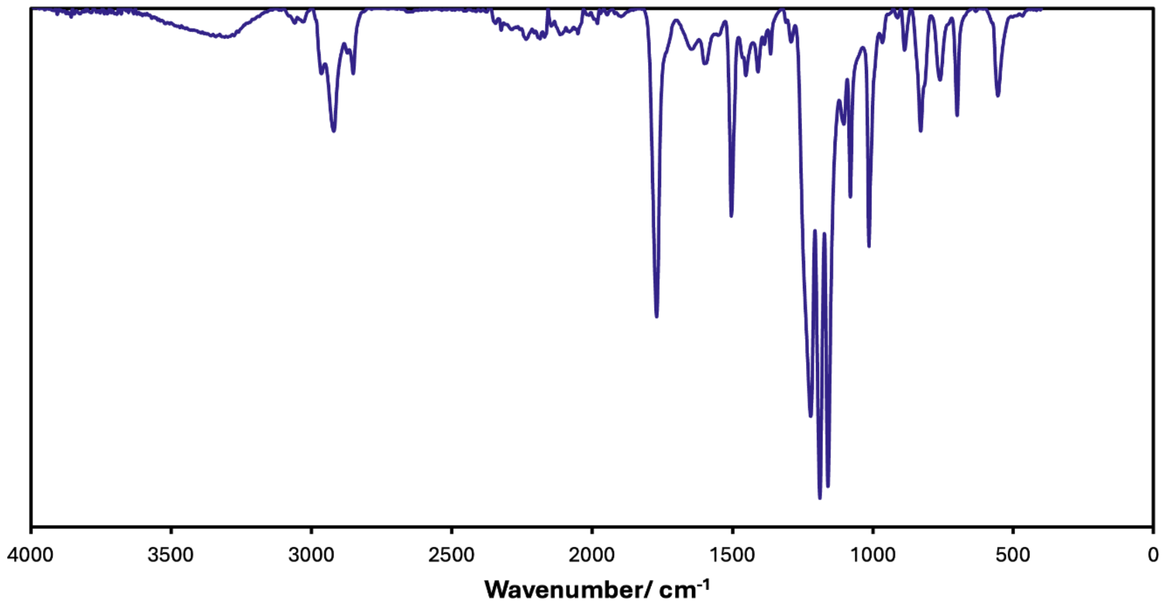

Figure S14. FTIR of PC/ABS air vent.

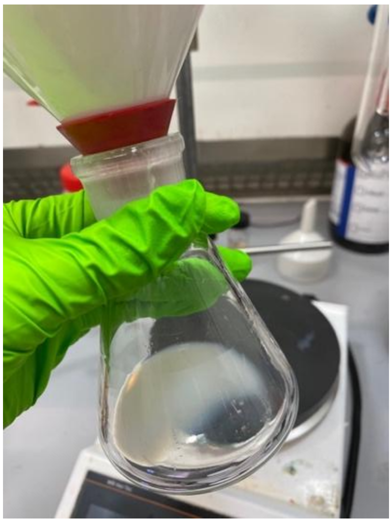

Figure S15. Result of filtering post-acetolysis PC/ABS mixture.

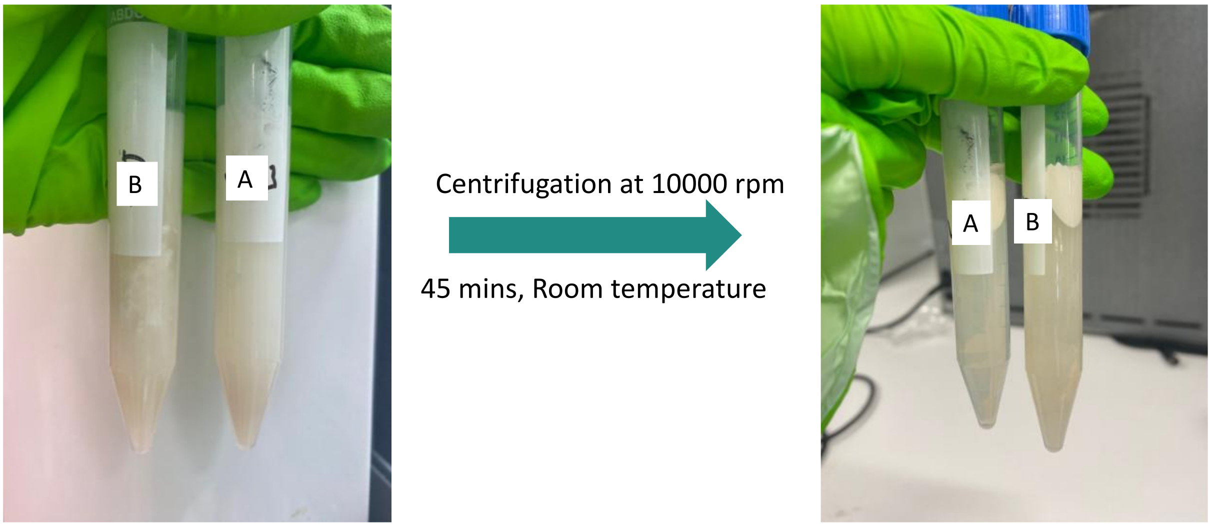

Figure S16. Sample A: post acetolysis PC/ABS was diluted with 7 ml of acetic acid. Sample B: post acetolysis PC/ABS sample was diluted with 6 ml of acetic acid and 1 ml of water. The tubes were spun at 10000 rpm for 45 minutes at room temperature.

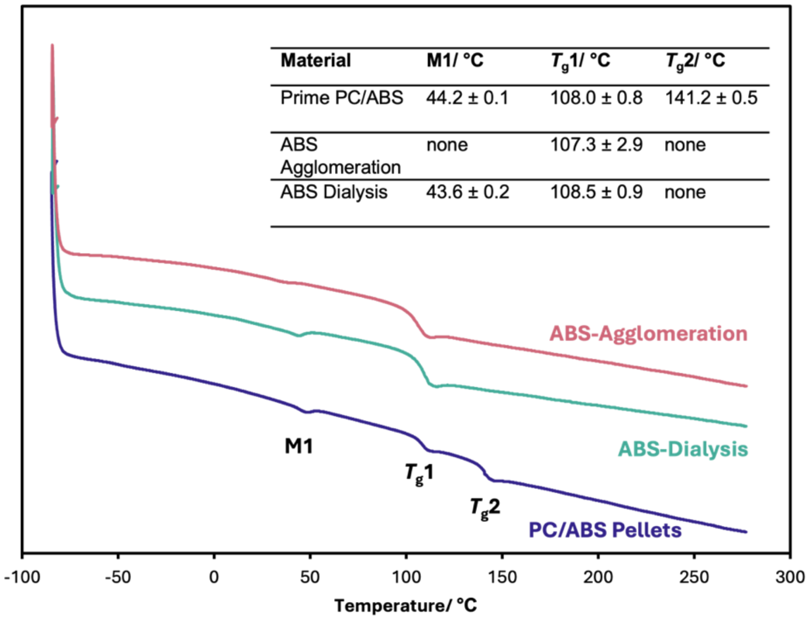

Figure S17. DSC results of PC/ABS pellets and the recycled ABS recovered using dialysis and agglomeration.

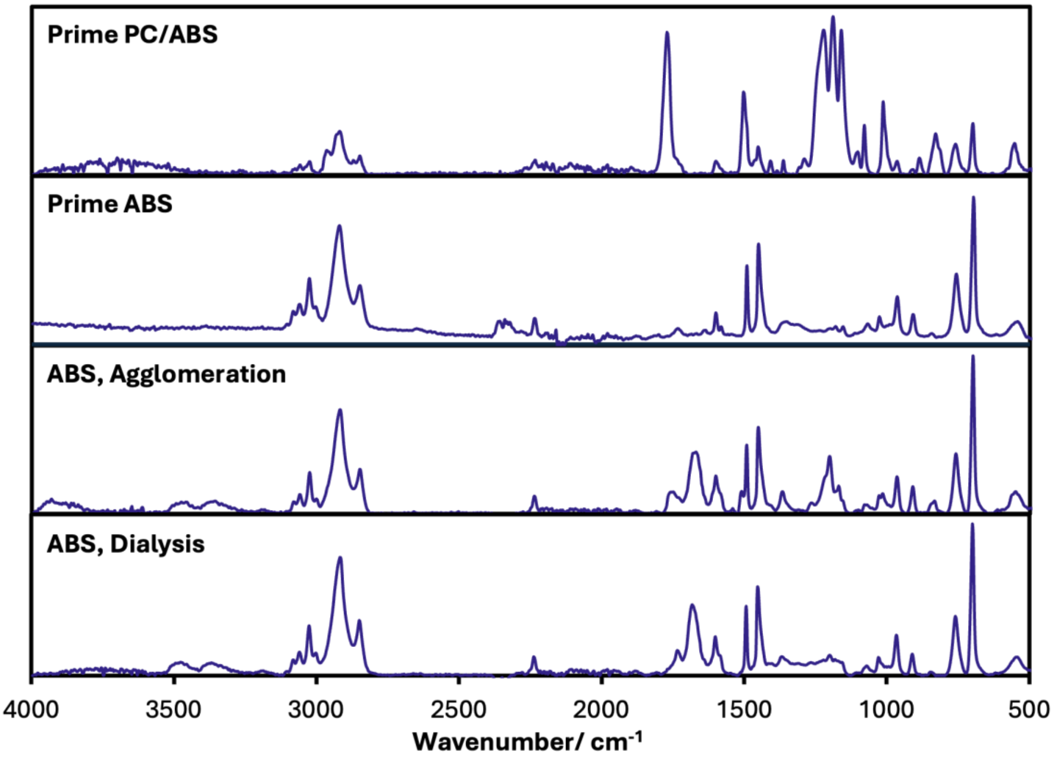

Figure S18. FTIR spectra comparing virgin PC/ABS and ABS to post acetolysis recycled ABS.

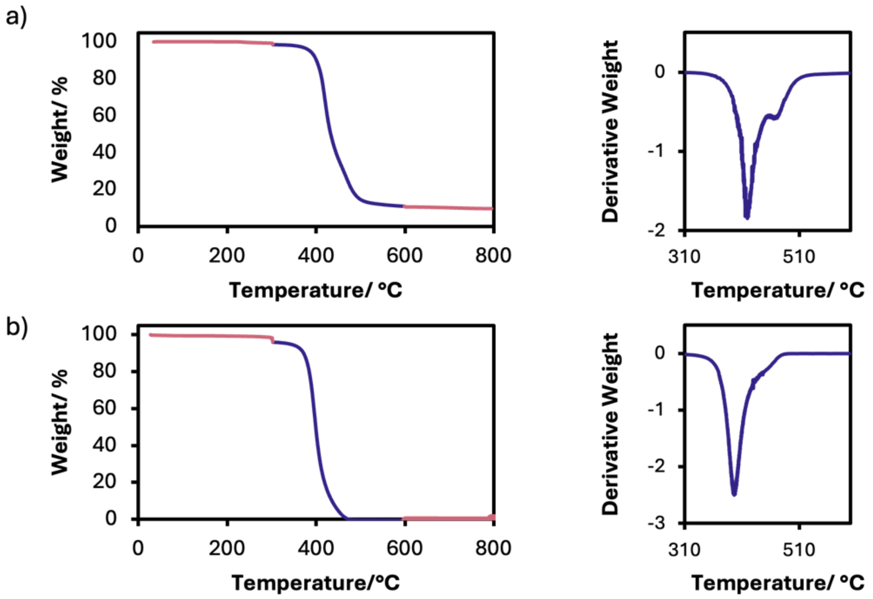

	Onset of degradation	Derivative Peak 1	Derivative Peak 2
Virgin PC/ABS	402.4 ± 1.0	419.6 ± 3.6	471.9 ± 1.5
Recycled ABS	376.2 ± 3.3	397.5 ±0.9	None

Figure S19. TGA data of a) ABS separated using dialysis compared to b) virgin PC/ABS.

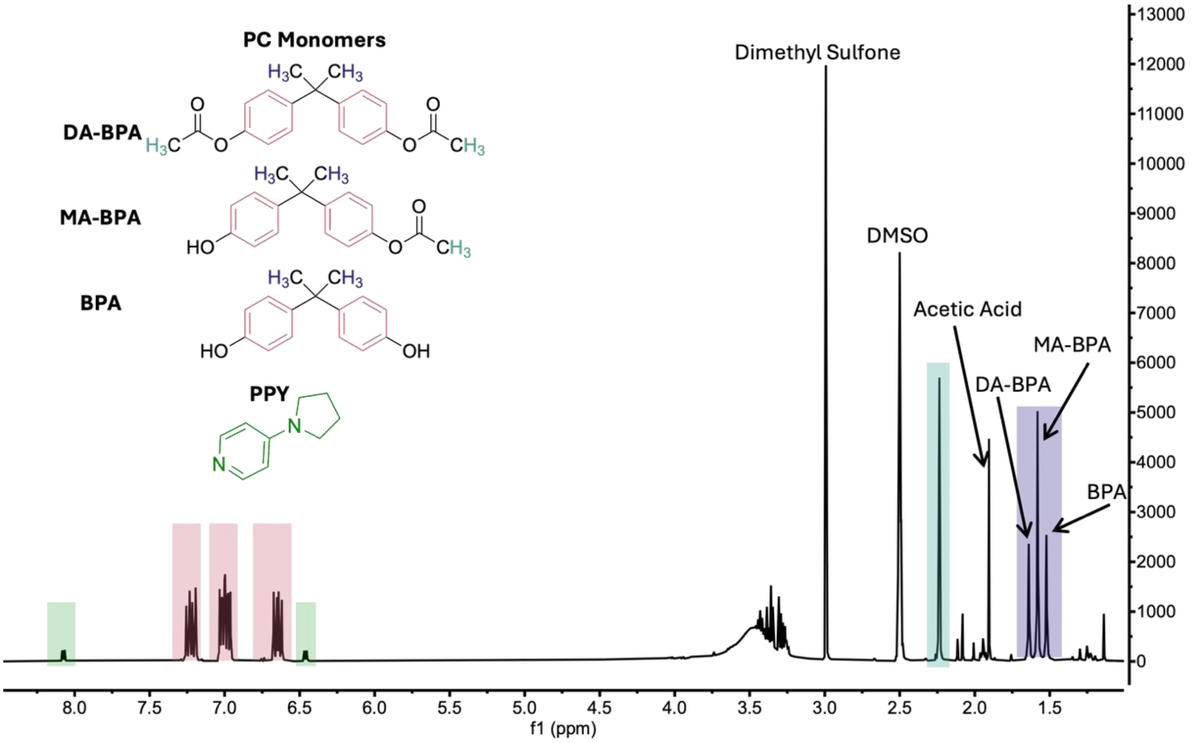

Figure S20. Oil containing monomers isolated using dialysis quantified using a known amount of dimethyl sulfone in DMSO-*d6*.

References

[44] Y. Zhang, C. Peng, G. Wang, X. Huang and Y. Yu, Hydroxyl-Free Epoxy Thermoplastics from Active Esters with Low Dielectric Constant and Water Sorption, *ACS Appl. Electron. Mater.*, **2024**, *6*, 4578–4586.

[45] S. Tshepelevitsh, A. Kütt, M. Lõkov, I. Kaljurand, J. Saame, A. Heering, P. G. Plieger, R. Vianello, I. Leito, "On the Basicity of Organic Bases in Different Media" *European Journal of Organic Chemistry* **2019**, *2019*, 6735–6748.

[46] C. Mao, Z. Wang, P. Ji, J.-P. Cheng, "Is Amine a Stronger Base in Ionic Liquid Than in Common Molecular Solvent? An Accurate Basicity Scale of Amines" *J. Org. Chem.* **2015**, *80*, 8384–8389.

[47] B. Lenarcik, P. Ojczenasz, "The influence of the size and position of the alkyl groups in alkylimidazole molecules on their acid-base properties" *Journal of Heterocyclic Chemistry* **2002**, *39*, 287–290.

[48] E. Marzocchi, S. Grilli, L. Della Ciana, L. Prodi, M. Mirasoli, A. Roda, "Chemiluminescent detection systems of horseradish peroxidase employing nucleophilic acylation catalysts" *Analytical Biochemistry* **2008**, *377*, 189–194.

[49] A. Toulmin, J. M. Wood, P. W. Kenny, "Toward Prediction of Alkane/Water Partition Coefficients" *J. Med. Chem.* **2008**, *51*, 3720–3730.
